# Phase‐Change Assembling Nanostructures Synergistically Potentiate Tumor Radiosensitivity by Reducing the Stemness of Cancer Stem‐Like Cells

**DOI:** 10.1002/EXP.20250179

**Published:** 2026-02-11

**Authors:** Yuanfang Chen, Xueyin Hu, Ze Hu, Yuwei Yang, Changfen Bi, Guangyou Shi, Lumeng Zhang, Wenqing Xu, Shuqin Li, Luntao Liu

**Affiliations:** ^1^ State Key Laboratory of Advanced Medical Materials and Devices Tianjin Key Laboratory of Radiation Medicine and Molecular Nuclear Medicine Tianjin Institutes of Health Science Institute of Radiation Medicine Chinese Academy of Medical Sciences & Peking Union Medical College Tianjin P. R. China; ^2^ Department of Pathology and Genomic Medicine Sidney Kimmel Cancer Center Thomas Jefferson University Philadelphia Pennsylvania USA

**Keywords:** cancer stem‐like cells, radiosensitization, deep delivery, improved tumor hypoxia, photothermal effect

## Abstract

Cancer stem‐like cells (CSCs) within deep tumors are a fundamental contributor to radiotherapy (RT) resistance due to their pronounced stemness resulting in unique unlimited self‐renewal and differentiation capabilities. Alleviating hypoxic microenvironment of deep tumors to attenuate the stemness of CSCs remains a significant challenge, as the dense extracellular matrix (ECM) severely restricts oxygen diffusion into deep tumors. Herein, a nano‐delivery particle (AMPM) is constructed to improve ECM permeability for deep tumor oxygen and radiosensitizer delivery. Natural fatty acid low‐melting eutectic mixtures are employed as phase change materials (PCM) to encapsulate thermoresponsive self‐assembled micelles, O_2_ pre‐saturated perfluoropentane, and nitroimidazole sensitizers (metronidazole, MTZ), with the goal of enhancing RT. Under 808 nm light irradiation, PCM acts as a temperature‐sensitive gatekeeper that undergoes solid‐to‐liquid phase transition under mild hyperthermic conditions (40°C), precisely controlling the release of MTZ and oxygen. Additionally, this design enhances the ECM permeability of the tumor, facilitating the delivery of oxygen and MTZ to deep‐seated tumors. In TNBC (triple‐negative breast cancer) mouse models, the combination of oxygen and MTZ effectively reverses radioresistance caused by hypoxic tumor microenvironment and CSCs, while significantly enhancing the efficacy of RT. Combination treatment with AMPM and RT (4 Gy) achieves a tumor inhibition rate of 91.2%, substantially surpassing high‐dose RT alone (12 Gy, 52.1% inhibition). This study presents an innovative sensitization strategy with considerable clinical application potential for radiosensitization.

## Graphical abstract

A eutectic phase‐change mixture of natural fatty acids was introduced to serve as a gating material for the near‐infrared‐triggered precise release of oxygen and nitroimidazole radiosensitizer in tumors. The stemness of cancer stem‐like cells was shown to be significantly reduced through the delivery of oxygen to deep tumors. The combination of oxygen with nitroimidazole radiosensitizers markedly potentiates tumor radiosensitivity and inhibits metastasis.

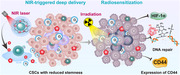

## 1 Introduction

Breast cancer is the most common malignant tumor among women. Triple negative breast cancer (TNBC), a subtype lacking expression of estrogen receptor (ER), progesterone receptor (PR), and human epidermal growth factor receptor 2 (HER‐2) [1], has limited treatment options due to a lack of biomarkers or drug targets [2]. This leads to aggressive tumors with high metastasis potential, poor prognosis, and recurrence rates [3, 4]. Radiotherapy (RT), as one of the most crucial treatment approaches for malignant tumors, participates in different treatment stages of approximately 70% tumor patients [5] which improves the local control of inoperable breast cancer and metastatic lesions [6, 7]. However, the 5‐year recurrence rate of advanced cancer patients who received RT alone without surgery is as high as 60%–70% [8, 9]. This is because the inherent and acquired tumor radioresistance severely limits the efficiency of RT and leads to treatment failure [10, 11, 12]. Therefore, overcoming RT resistance to enhance RT efficacy and reduce damage to normal tissues is a key problem that needs to be addressed. Cancer stem‐like cells (CSCs) are a distinctive subgroup of cancer cells with the ability of continuous self‐renewal and differentiation, playing a vital role in tumorigenesis, development, metastasis, and recurrence [13, 14]. Compared with tumor cells, CSCs exhibit stronger RT resistance and are widely considered to be the root cause of tumor radioresistance [15, 16, 17, 18]. Research on the relationship between CSCs and RT resistance has made an important breakthrough in recent years. A study published in Cell Metabolism in 2024 found that glioma stem cell marker ALDH1A3 induced lactate accumulation and modified DNA repair protein XRCC1 by interacting with glycolysis rate‐limiting enzyme PKM2, leading to RT resistance. This work provides an argument for the mechanism of CSCs and RT resistance [18].

Since Thomlinson and Gray discovered hypoxic cells in solid tumors and linked them to poor RT outcomes, tumor hypoxia has aroused great concern in radiobiology and oncology [19, 20, 21, 22]. Studies have pointed out that CSCs are mostly located in the center of the tumor mass, which is characterized by hypoxia and low pH [23]. CSCs can promote their expansion by reducing cellular radiation damage and modifying their gene expression profiles, further aggravating RT resistance [24, 25, 26]. Hypoxia has been confirmed as a critical factor in enhancing the stemness and phenotype of CSCs, and is considered to be a major obstacle to achieving radiosensitization [27, 28]. Liu's group reported that in situ cell membrane self‐assembly radiosensitizer (CA‐Pt), by targeting carbonic anhydrase IX (CAIX), enriches CSCs improves hypoxic microenvironment, and inhibits stemness maintenance. CSCs‐based radiosensitization remains a challenge because deep tumor hypoxic microenvironment limits drug delivery and therapeutic efficacy [24]. Therefore, efficient delivery of oxygen to deep tumor regions is of great significance for reducing the stemness of CSCs.

In the current domain of nanomedicine, personalized and refined nanomedicines are employed to ameliorate the hypoxic environment of tumors [29], encompassing enhancing the oxygen concentration within tumors [30], altering the metabolic pathways of tumors to reduce reliance on hypoxic conditions, and normalizing tumor blood vessels [31, 32, 33, 34]. The most straightforward approach is to directly convey oxygen to the tumor site. Nevertheless, upon the arrival of oxygen at the tumor region, the dense ECM impede the deep delivery of oxygen [33]. The ECM is comprised of fibronectin, collagen, and various binding proteins, which collectively establish a complex three‐dimensional physical barrier within tumor tissue [35, 36]. This dense structure creates resistance to molecular diffusion from the perivascular region to distal cells; even O_2_ molecules are limited in their diffusion, reaching only 100–200 µm from the vascular source [37, 38, 39]. The dense ECM of tumors results in poor permeability. Hence, the bottleneck issue in oxygen delivery to the tumor site is the poor permeability within the tumor tissue, making it arduous to reach the central area of solid tumors. Studies have confirmed that the photothermal effect can denature tumor collagen, improve the permeability of tumor tissue, reduce the hindrance of the dense ECM to nanomedicines, and facilitate the deep penetration of nanomedicines [40, 41].

**SCHEME 1:**
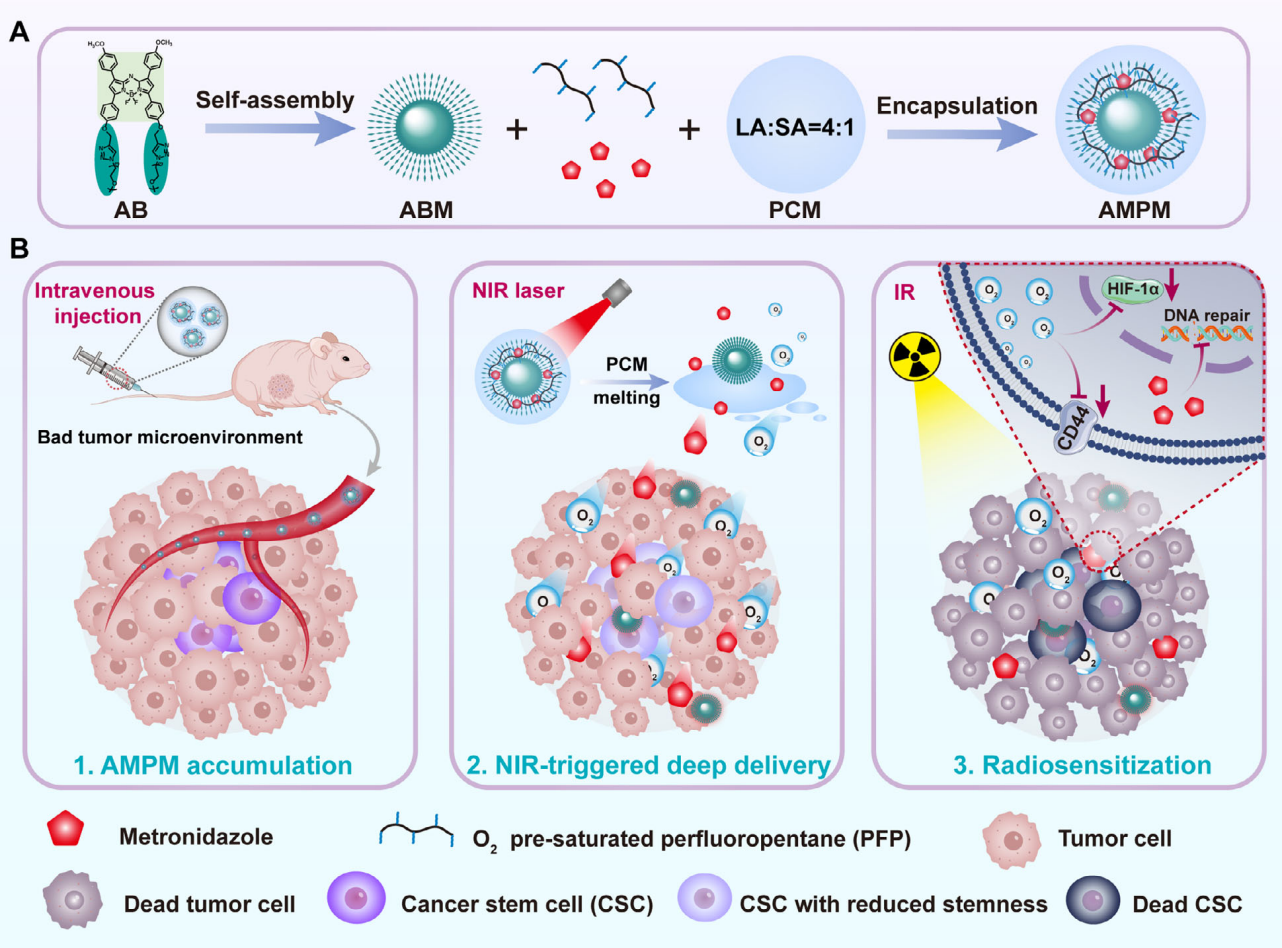
Schematic diagram of the intelligent responsive RT sensitization platform enhancing the RT effect on deep tumors. (A) Components and formation processes of AMPM. (B) AMPM responds to NIR for the deep delivery of oxygen and MTZ, aiming to ameliorate tumor hypoxia, reduce the stemness of CSCs and inhibit the repair of DNA damage caused by RT, ultimately achieving radiosensitization.

To overcome hypoxia and the ECM barrier limit the efficacy of radiosensitizer in CSC‐rich tumor cores, this study developed a thermosensitive nanoplatform (AMPM), whose core components include temperature‐responsive PCM, NIR‐driven photothermal micelles (ABM), nitroimidazole‐based radiosensitizer (MTZ) [42]. and highly efficient oxygen‐carrying PFP (Scheme 1A). The PCM are a eutectic mixture formed by two natural fatty acids (Lauric acid: Stearic acid = 4: 1), which can undergo solid‐liquid phase transition in response to temperature changes. The photothermal effect of ABM triggers the phase transition of PCM and simultaneously lead to the inactivation of ECM collagen and increased tumor permeability, thereby facilitating the delivery of oxygen and MTZ to deep tumors. Oxygen effectively alleviates the hypoxic characteristics of deep tumors, reduces the stemness of CSCs, and inhibits the expression of HIF‐1α (Scheme 1B). TMZ enhances the lethal effect of RT on tumor cells by inhibiting DNA damage repair function. In the mouse TNBC model, AMPM efficiently reduced the expression of CD44 in CSCs, inhibited HIF‐1α, and suppressed DNA damage repair in tumor cells, demonstrating a tumor inhibition rate of 91.2%, which is significantly higher than that of the high‐dose RT (12 Gy, tumor inhibition rate of 52.1%) alone. Therefore, AMPM can successfully alleviate tumor RT resistance and provide valuable guidance for the development of high‐performance radiosensitizers.

## 2 Results and Discussions

### 2.1 Preparation and Performance Characterization of AMPM

**FIGURE 1:**
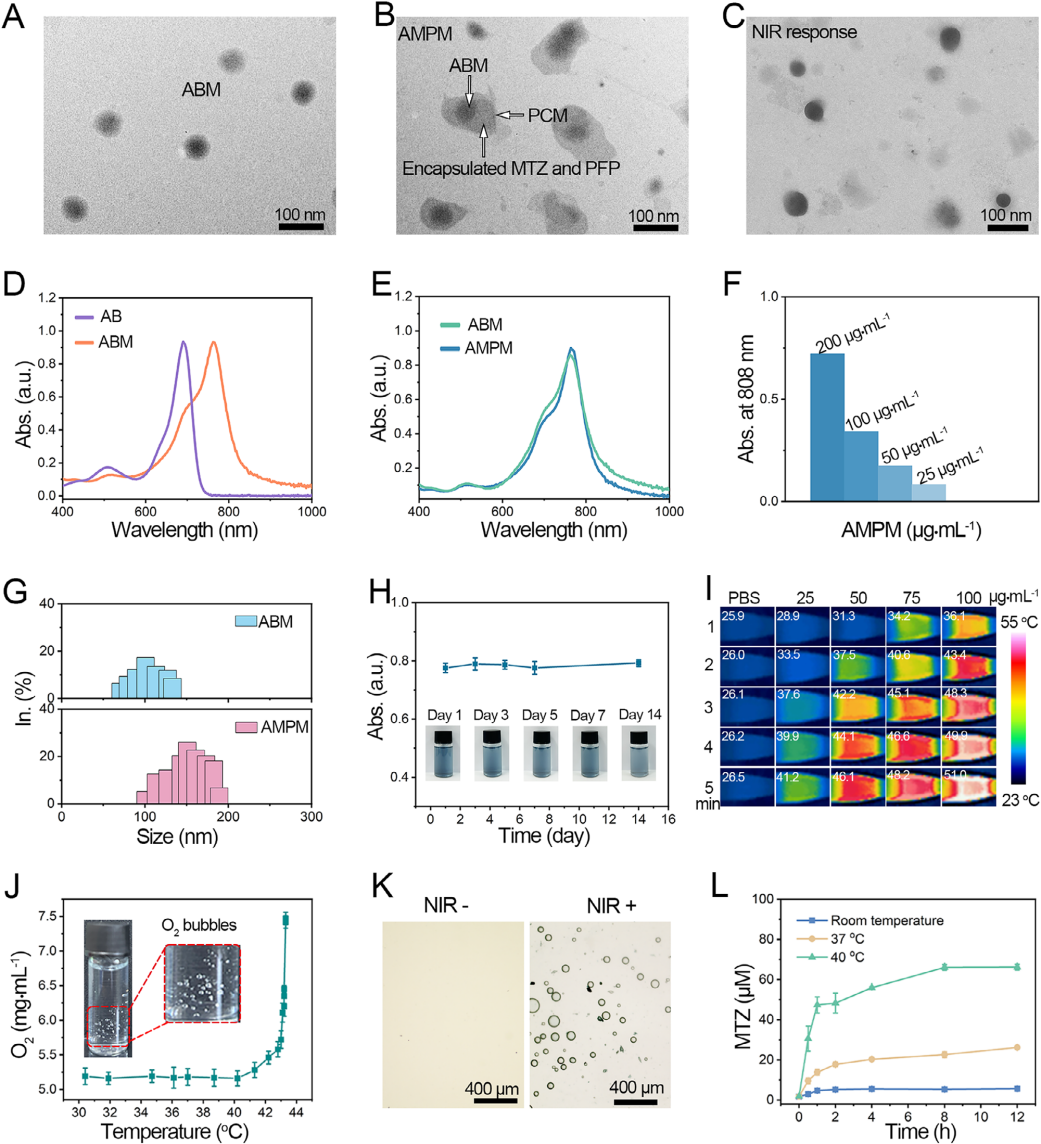
Characterization of AMPM. (A) TEM image of ABM. (B) TEM image of AMPM. (C) TEM image of AMPM after NIR (808 nm, 0.5 W•cm^−2^ for 5 min) irradiation. (D) UV/vis absorption spectra of monomer (10 µm, purple line) in methanol, ABM (50 µm, orange line) in water at room temperature. (E) UV/vis absorption spectra of ABM (50 µg•mL^−1^, green line), AMPM (100 µg•mL^−1^, blue line) in PBS at room temperature. (F) UV/vis absorbance at 808 nm for different concentrations (200 µg•mL^−1^, 100 µg•mL^−1^, 50 µg•mL^−1^, 25 µg•mL^−1^) of AMPM in PBS. (G) The size distribution of ABM and AMPM (50 µg•mL^−1^) PBS solution at 37°C tested by dynamic light scattering (DLS). (H) UV/vis absorbance of the maximum absorption peak (769 nm) of AMPM (100 µg•mL^−1^) in PBS within 14 days, inset images show the optical images of AMPM at different time points. (I) Heating images of AMPM solution at different concentrations (0, 25, 50, 75, and 100 µg•mL^−1^) under 808 nm (0.5 W•cm^−2^) within 5 min. The specific temperature is shown in the top left corner of the picture (°C). J) O_2_ concentration curves of oxygen release from AMPM at increased temperature (mean ± SD, *n* = 3). The inset figure is the optical image of AMPM after 808 nm irradiation (0.5 W•cm^−2^) for 3 min. (K) Photographs of bubbles generation of AMPM solution before and after laser irradiation (808 nm, 0.5 W•cm^−2^ for 3 min) [NIR^−^] represented without 808 nm laser irradiation; [NIR^+^] represented with 808 nm laser irradiation. (L) Curves of drug release from AMPM over 12 h at room temperature, 37°C, and 40°C (mean ± SD, *n* = 3).

Amphiphilic aza‐BODIPY (AB) were synthesized via Cu^I^‐catalyzed “click” reaction (Scheme S1 and Figures S1–S8) and self‐assembled into 50 nm nanoparticle micelles (ABM; Figure 1A). ABM, PFP, and MTZ were encapsulated in photothermal responsive PCM to form ∼120 nm AMPM nanoparticles, which release ABM and MTZ upon PCM phase transition under heating (Figure 1B,C). Spectral characterization showed AB had a UV/vis absorption maximum at 679 nm (Figure 1D), while self‐assembled ABM exhibited a red‐shifted absorption at 769 nm, ensuring that ABM could produce a photothermal effect under 808 nm irradiation. AMPM showed no significant spectral difference from ABM (Figure 1E). Concentration‐dependent UV/vis spectra of AMPM in PBS revealed decreasing 808 nm absorption with lower concentrations (Figure S9 and Figure 1F), and no disassembly even at 25 µg•mL^−1^, confirming dynamic stability. We performed differential scanning calorimetry (DSC) analysis on both the PCM and AMPM nanosystems (Figure S10). The DSC curves showed a distinct endothermic peak at 40°C, which corresponds to the melting point of the LA:SA mixture. AMPM still exhibited a clear endothermic peak at 40.5°C, indicating that the encapsulation process did not alter the melting point of PCM. Dynamic light scattering (DLS) showed ABM and AMPM had hydrodynamic diameters of 90 and 135 nm, respectively (Figure 1G). The size distribution of AMPM after NIR irradiation is shown in Figure S11, which remains a range of 40–350 nm. Stability tests showed no significant change in the absorbance of AMPM at 808 nm within 14 days (Figure 1H). Additionally, the hydrodynamic diameter of AMPM remained relatively stable in PBS and FBS over 72 h (Figure S12), demonstrating that AMPM possesses favorable physiological stability.

The photothermal effect of AMPM is crucial for the responsive release of drugs and oxygen. Consequently, the photothermal effect of AMPM with different concentrations was recorded under 808 nm laser irradiation at different power densities (Figures S13 and S14). The temperature of the AMPM solution is positively correlated with the laser intensity and the concentration of the solution. Heating images of AMPM solution at different concentrations under 808 nm within 5 min were shown in Figure 1I. The AMPM solution demonstrated a significant temperature increase with increasing concentration and time. At 100 µg•mL^−1^, AMPM solution reached 51°C from 25°C after 5 min laser irradiation. With PCM's phase transition temperature at 40°C, this facilitates PCM's solid‐liquid transition for oxygen and drug release. Periodic laser on/off‐induced heating/cooling cycles showed no significant temperature drop (Figure S15) or UV/vis spectral changes (Figure S16), confirming AMPM's excellent photostability. Consistent UV‐Vis spectra of AMPM after 808 nm laser irradiation on day 1 and 30 (Figure S17) further validated the photothermal stability of ABM, the source of AMPM's photothermal properties.

Perfluorocarbon (PFP), a clinically approved agent by European Medicines Agency and a biocompatible ultrasound contrast agent with an initial boiling point of 29°C (elevated to 40–50°C in vivo due to blood pressure) [43, 44], enables oxygen release from AMPM [45]. AMPM released oxygen gradually above 40°C and sharply above 43°C (Figure 1J), due to PFP boiling (exceeding its temperature threshold) and releasing pre‐saturated oxygen (inset shows oxygen release). Under 808 nm laser irradiation, optical microscopy revealed significant bubble formation in AMPM after 5 min (Figure 1K), confirming laser—triggered oxygen release. Subsequently, we investigated the drug release process from AMPM following laser irradiation. Initially, a standard curve for MTZ concentration versus absorbance was established using UV‐Vis absorption spectroscopy (Figure S18). Drug release assays (Figure 1L) showed minimal MTZ release at room temperature, slight release at 37°C and rapid release at 40°C (30.5 µm within 30 min, plateauing thereafter). 12 h dialysis yielded 5.6, 26.2, and 66.2 µm MTZ at room temperature, 37°C, and 40°C, respectively, confirming temperature‐responsive release. NIR‐triggered release (Figure S19) showed no MTZ release at 0 W·cm^−2^; 0.2 W·cm^−2^ induced release at 4 min (17.6 µm), while 0.5 W·cm^−2^ accelerated release to 2 min (18.5 µm), indicating higher laser power shortens PCM phase transition and MTZ release time.

### 2.2 Evaluation of In Vitro Radiosensitization of AMPM

**FIGURE 2:**
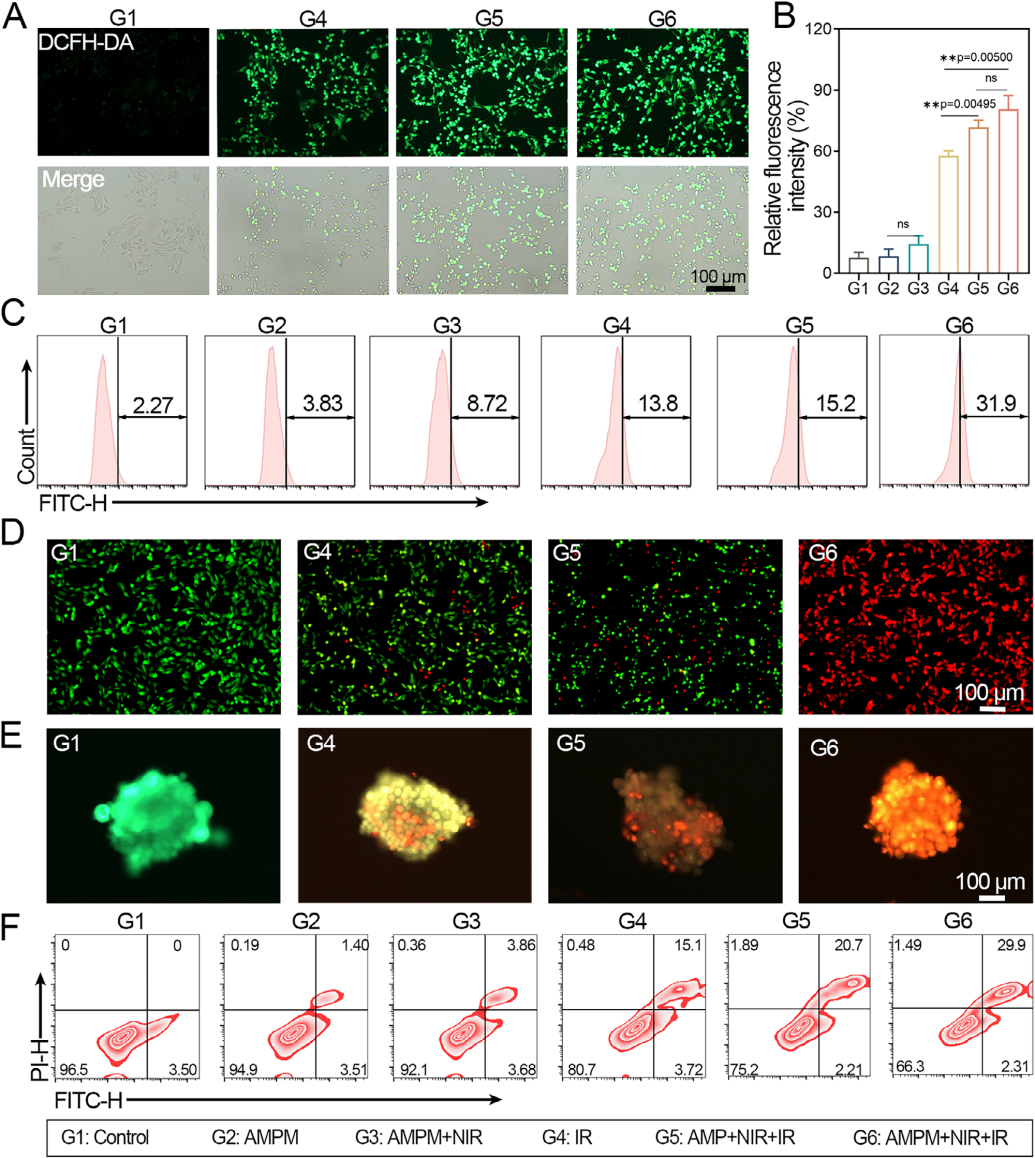
In vitro antitumor evaluation of AMPM. (A) Fluorescence image of MDA‐MB‐231 cells treated with G1, G4, G5, and G6 using DCFH‐DA staining (*λ*_ex_ = 488 nm, *λ*_em_ = 500–540 nm). IR: Cells were irradiated with *γ*‐ray irradiator (0.88 Gy min^−1^) at 4 Gy), NIR: Cells were treated with 808 nm irradiation at 0.5 W•cm^−2^ for 3 min. (B) Quantitative analysis of fluorescence based on (A) (mean ± SD, *n* = 3, **p < 0.01, ns = non‐significant). (C) Flow cytometry ROS analysis of MDA‐MB‐231 cells treated with groups of G1–G6 at 24 h (numbers represent ROS level). (D) Fluorescence images of MDA‐MB‐231 cells costained with calcein AM (live cells, green fluorescence) and PI (dead cells, red fluorescence) treated with G1, G4, G5, and G6. (E) Fluorescence images of MDA‐MB‐231 cells spheroids costained with calcein AM and PI treated with G1, G4, G5, and G6. (F) Apoptosis ratios of MDA‐MB‐231 cells cultured treated with different groups (G1–G6).

To evaluate the radiosensitization effect of AMPM in the responsive delivery of MTZ and oxygen, we assessed the cytotoxicity of AMPM on tumor cells by establishing various control groups. ABM and oxygen‐saturated PFP was encapsulated with PCM to create the control material (AMP). The UV/vis absorption spectra, morphology, and size was analyzed in Figure S20. In comparison to AMPM, the UV/vis absorption spectra of AMP exhibited no notable variance (S20A), with an average size of 122 nm (S20B‐C). Radiation induces cellular damage both directly (DNA injury) and indirectly (via ROS generated by ionizing radiation). MDA‐MB‐231 cells cultured under hypoxia were treated differently, and ROS levels were detected using the DCFH‐DA probe. Compared with the control group (G1), the single administration group (G2) showed no significant difference in ROS generation in Figure 2A and Figure S18. Under 808 nm laser irradiation, AMPM (G3) produced a small amount of ROS, with no significant difference from G2 (Figure S21). Under 4 Gy irradiation, the G4 group exhibited ROS production, confirming radiation‐induced ROS generation. Compared with G4, AMP (containing oxygen‐saturated PFP) significantly increased ROS levels under irradiation (statistically different from G4). AMPM (also with oxygen‐saturated PFP) showed notably enhanced ROS fluorescence under 4 Gy irradiation, with no significant difference from G5. Though AMPM contains MTZ, this experiment provided no evidence that MTZ plays a key role in ROS elevation.

To assess the impact of oxygen concentrations on treatment efficacy, cells were cultured under hypoxia and treated with AMPM encapsulating varying doses of PFP. PFP doses ≥200 µL slightly reduced cell viability, with 500 µL decreasing survival to 62% (Figure S22). Therefore, our optimized AMPM formulation maximizes oxygen loading while minimizing cytotoxicity. Figure 2B shown the quantitative analysis of fluorescence according to Figure 2A. Flow cytometry revealed that under hypoxia, 4 Gy irradiation significantly increased ROS levels (Figure 2C), which was further enhanced by oxygen supplementation during irradiation. Cytotoxicity assays (Figure S23) showed that AMPM alone (G2/G3) was non‐toxic at 100 µg/mL with laser irradiation. While 4 Gy irradiation alone (G4) reduced viability to 75.2%, combining irradiation with oxygen (G5) increased cytotoxicity. The combination of oxygen, radiosensitizer (AMPM), and irradiation (G6) yielded optimal efficacy, reducing cell viability to 26.3%.

Cytotoxicity across treatment groups was assessed in cells and multicellular spheroids (MCSs) (Figure 2D,E and Figure S24). Groups G2 and G3 showed no significant cytotoxicity versus G1. G4 (4 Gy irradiation alone) induced cell death in both models. G5 (AMP with O_2_‐saturated PFP + 4 Gy) enhanced cell killing in hypoxic cultures. G6 (AMPM with O_2_‐saturated PFP, MTZ + 4 Gy) exhibited the strongest cytotoxic effect in both cells and MCSs, attributable to MTZ stabilizing radiation‐induced DNA damage and inhibiting repair. Flow cytometry analysis of apoptosis (Figure 2F) corroborated these findings: G2/G3 induced minimal apoptosis versus G1; 4 Gy irradiation (G4) significantly increased apoptosis versus Control; oxygen supplementation (G5) further increased apoptosis to 22.91% versus G4 (18.82%); and G6 demonstrated the highest apoptosis (32.21%), demonstrating pronounced radiosensitization at the cellular level.

**FIGURE 3:**
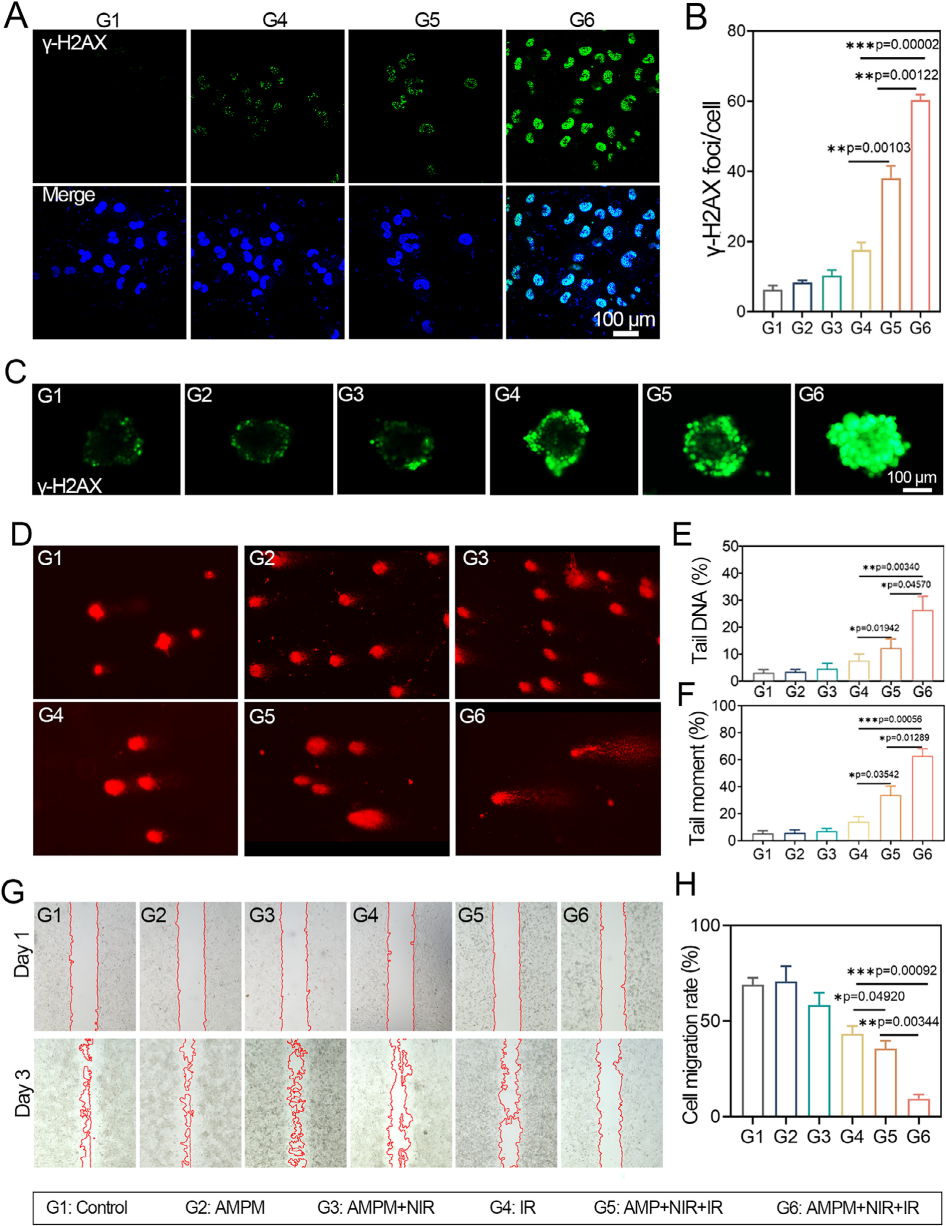
Evaluation of AMPM's effects on tumor cell DNA damage and migration inhibition. (A) CLSM microscopy observation of γ‐H2AX immunofluorescence of MDA‐MB‐231 cells treated with G1, G4, G5, and G6. (B) Quantitative analysis of γ‐H2AX foci from (A). Results are expressed as the mean ± SD of 20 replicates. (C) Immunofluorescent γ‐H2AX staining of MDA‐MB‐231 cells spheroids treated with G1–G6. D) Typical comet assay images of MDA‐MB‐231 cells treated with groups of G1–G6. Quantitative analysis of tail DNA (E) and olive tail moment (F) from Figure 3D. (G) Evolution of MDA‐MB‐231 cells wound healing treated with various treatments. (H) Cell migration rate calculated from (G). A *p* value of *p < 0.05, **p < 0.01, and ***p < 0.001 was considered statistically significant.

γ‐H2AX, a key marker of radiation‐induced DNA double‐strand breaks (DSBs) [46], was used to validate AMPM's radiosensitization. Consistent with reports of peak foci formation at 0.5 h post‐irradiation [47], hypoxic MDA‐MB‐231 cells from each group were assessed for γ‐H2AX foci (green) 30 min after 4 Gy irradiation; nuclei were counterstained with DAPI (blue) (Figure 3A and Figure S25). Similar to the G1, groups G2 and G3 showed no γ‐H2AX foci. Foci were detected in G4 (4 Gy alone), confirming radiation‐induced DNA damage. Enhanced foci intensity in G5 (AMP with O_2_‐saturated PFP + 4 Gy) versus G4 suggested improved radiosensitivity via hypoxia alleviation. The strongest foci signal and DNA damage occurred in G6 (AMPM with O_2_‐saturated PFP and MTZ + 4 Gy), attributed to combined oxygen delivery and metronidazole's stabilization of radiation‐induced DNA damage, inhibiting repair. Statistical analysis (Figure 3B) confirmed that AMPM‐released oxygen and MTZ significantly increased DSBs. MDA‐MB‐231 multicellular spheroids (MCSs) were established to model solid tumors in vitro (Figure 3C). Post‐treatment γ‐H2AX analysis revealed that G2/G3 showed no significant DNA damage versus control (G1). In G4 (4 Gy irradiation), Peripheral γ‐H2AX foci indicated radiation‐induced DNA damage, while hypoxic cores remained undamaged. Enhanced γ‐H2AX fluorescence in G5 (AMP with O_2_‐saturated PFP + 4 Gy), suggesting partial hypoxia alleviation by NIR‐triggered oxygen release improved radiosensitivity. MTZ can inhibit the repair of damaged DNA and thus act as a radiosensitizer. When loaded into AMPM, MTZ is released in response to NIR irradiation, which enhances the efficacy of RT and exhibits a favorable DNA—damaging effect. Strongest γ‐H2AX signals in G6 (AMPM with O_2_‐saturated PFP and MTZ + 4 Gy), demonstrating optimal DNA damage via NIR‐activated oxygen/sensitizer co‐delivery to overcome radioresistance in MCSs.

Irradiation‐induced DNA double‐strand breaks (DSBs) cause DNA unwinding and migration under electrophoresis, forming a “comet tail”‐assessed via comet assay using Olive tail moment and tail DNA content as markers [48] Since the cell shape resembles a comet after electrophoresis, this experiment is referred to as the comet assay [49]. Comet assay quantified DNA damage using Olive tail moment and tail DNA (Figure 3D,E). Compared to control, G2/G3 groups showed no significant damage, confirming AMPM (100 µg•mL^−1^ ± NIR) lacked genotoxicity. G4 (4 Gy) exhibited detectable tail moments, verifying radiation‐induced damage. G5 (AMPM + O_2_ + 4 Gy) demonstrated markedly elongated tails, indicating oxygen‐mediated radiosensitization. Maximal DNA fragmentation occurred in G6 (AMPM + O_2_/MTZ + 4 Gy) with peak tail moments, demonstrating synergistic DSB potentiation via NIR‐triggered oxygen/MTZ co‐release. Corroborating γ‐H2AX data, these results confirm that AMPM maximizes radiation‐induced DNA damage through dual oxygen/sensitizer delivery, providing mechanistic evidence for its radiosensitizing efficacy.

It has been reported that tumor resistance to radiation therapy can lead to tumor migration and recurrence [50, 51, 52]. Hypoxic tumor cell migration post‐treatment was assessed (Figure 3G). While G1and G2 showed comparable migration rates, G3 (AMPM + NIR) exhibited mild inhibition, suggesting partial oxygen‐mediated suppression. Radiation alone (G4, 4 Gy) reduced migration to 43.3%. This was further suppressed in G5 (AMP + O_2_ + 4 Gy; 35.6%) via hypoxia alleviation. Optimal inhibition occurred in G6 (AMPM + O_2_/MTZ + 4 Gy; 9.3%), attributed to dual oxygen delivery and MTZ‐induced DNA repair blockade. Statistical analysis confirmed significant advantage of G6 over G4/G5 (Figure 3H), demonstrating potent anti‐migratory effects.

### 2.3 Evaluation of In Vitro Radiosensitization of CSCs

**FIGURE 4:**
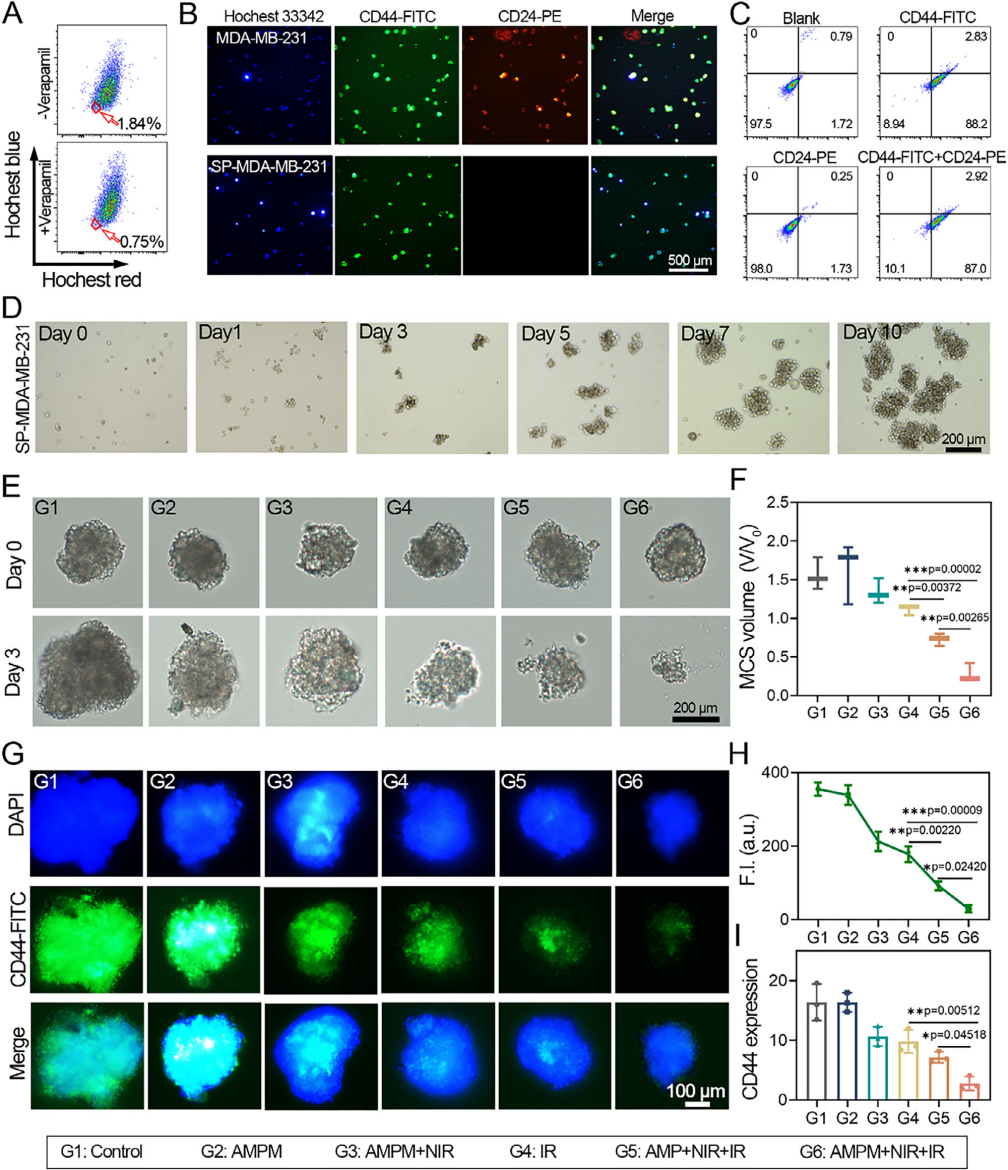
Evaluation of radiosensitization of AMPM to CSCs in vitro. (A) Representative images of flow cytometric sorting of SP cells in MDA‐MB‐231 cells. (B) Fluorescence images of differential expression of CD44 and CD24 in MDA‐MB‐231 parental cells and their suspended CSCs (SP‐MDA‐MB‐231 cells). (C) Flow cytometry analysis of differential expression of CD44 and CD24 in MDA‐MB‐231 parental cells and SP‐MDA‐MB‐231 cells. (D) Representative images of the tumor spheres formed by SP cells culturing in the ultra‐low attachment 6 well plates within 10 days. (E) Destructive effects of different treatments on hypoxic 3D spheroids of SP‐MDA‐MB‐231 cells. (F) Quantitative analysis of cell spheroid volume based on Figure 3E. (G) Representative fluorescence images of CD44 expression of SP‐MDA‐MB‐231 3D spheroids after 24 h treatment by various formulations. (H) Fluorescence quantification calculated from Figure 3G. (I) qPCR analysis of CD44 expression in SP‐MDA‐MB‐231 cells after 24 h in the presence of different. A *p* value of *p < 0.05, **p < 0.01, and ***p < 0.001 was considered statistically significant.

CSCs possess unlimited self‐renewal and differentiation capabilities [53]. In comparison to tumor cells, CSCs exhibit significantly enhanced radioresistance, which is considered as a fundamental reason of treatment resistance in tumors [54]. These CSCs are primarily located at the tumor center, a region characterized by hypoxia and low pH [13]. Therefore, penetrating deeply into tumors to influence stem cell differentiation is essential for overcoming radiation resistance. Research has demonstrated a close correlation between RT resistance and prognosis in breast cancer patients [55]. In 2003, Al‐Hajj et al. first isolated CSCs from malignant pleural effusions and metastatic sites in breast cancer patients [10], identifying their phenotype as CD44^+^/CD24^−/low^. This study investigates the effects of a nano‐delivery platform on the treatment of human breast cancer stem cells MDA‐MB‐231. The MDA‐MB‐231 CSCs were c isolated from MDA‐MB‐231 using Hoechst 33342‐based side population (SP) sorting via flow cytometry (Figure 4A). Fluorescence microscopy images (Figure 4B) demonstrate that sp‐MDA‐MB‐231 stem cells exhibit higher CD44 and lower CD24 expression than parental cells, with flow cytometry confirming their CD44^+^/CD24^−/low^ phenotype (Figure 4C). When cultured in serum‐free medium, SP‐MDA‐MB‐231 cells formed suspended spheres composed of cell aggregates after 24 h (Figure 4D). To investigate the stemness of stem cells, we performed low‐cell‐number tumorigenicity assays. Even at a low cell seeding density (1×10^3^ cells), measurable tumors were formed at axillary injection sites (Figure S26). These findings directly confirm the significant in vivo tumorigenic potential of sorted CD44^+^/CD24^−^ cells, further validating their stem cell properties. Over a period of 3 to 10 days, both the volume and number of the suspended spheres gradually increased. Following the passage of these suspended cells, cell spheres were subsequently established in ultra‐low attachment 96‐well plates.

Cell spheres responses to treatments are shown in Figure 4E. Compared to control (G1), groups G2‐G3 showed no significant cytotoxicity. While G4 (4 Gy) inhibited tumorsphere growth, G5 (AMPM + NIR + 4 Gy) induced significant cytotoxicity through NIR‐triggered oxygen release improving hypoxia and radiosensitivity. G6 (AMPM + NIR + 4 Gy) exhibited maximal suppression with 68.3% volume reduction (Figure 4F), attributed to synergistic oxygen/MTZ co‐delivery enhancing DNA damage stabilization. Quantitative analysis of tumor sphere volumes confirmed these observations. Evaluation of CD44 expression in tumor spheres showed G6 MCSs had the smallest size and lowest CD44 fluorescence (Figure 4G,H). Furthermore, the expression levels of cancer stem cell (CSC)‐related genes, including Sox‐2, Nanog, and Oct‐4, in the cell spheres are presented in Figure S27. Consistent with the expression pattern of CD44, the G6 group showed significantly lower levels of these stemness markers compared to the G4 and G5 groups. Quantitative PCR further revealed G6 markedly downregulated CD44, reducing the stemness of SP‐MDA‐MB‐231 cells.

These results demonstrate that AMPM acts as a potent radiosensitizer for CSCs, offering a novel strategy to overcome tumor radioresistance. Crucially, AMPM‐mediated reduction of CSC stemness significantly enhances therapeutic completeness. However, the molecular mechanisms governing AMPM's stemness modulation remain incompletely elucidated and may involve multifaceted signaling pathways. Additionally, complex CSC‐microenvironment interactions could influence AMPM efficacy. Future studies should address these mechanisms to optimize treatment paradigms.

### 2.4 Deep Tumor Penetration, In Vivo Biodistribution and Photothermal Effects Evaluation of AMPM

**FIGURE 5:**
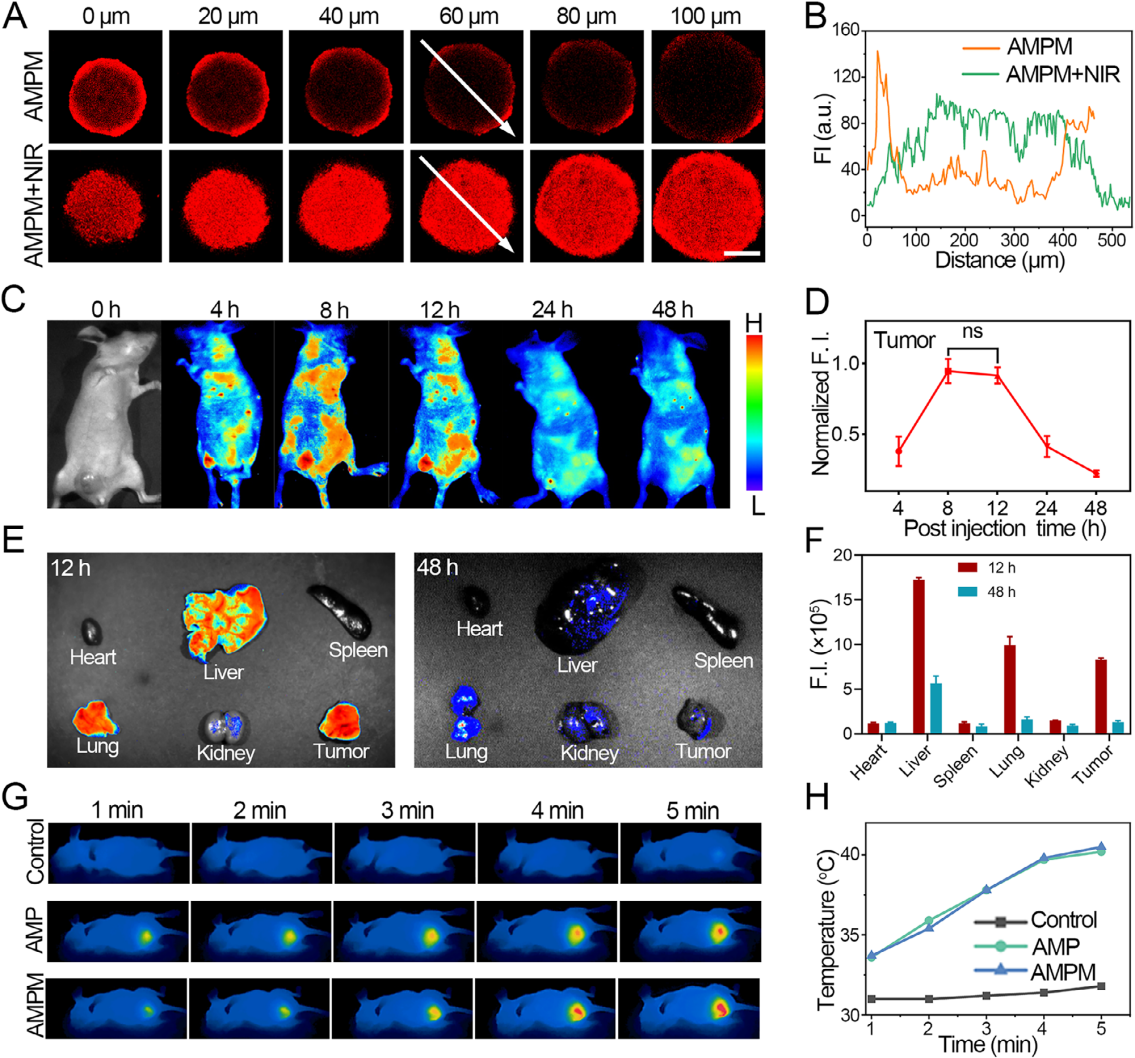
Evaluation of AMPM's in vitro deep tumor penetration, in vivo biological distribution, and photothermal effects. (A) Deep penetration of the Nile red‐labeled AMPM into MDA‐MB‐231 tumor spheroids using LSCM analysis (Scale bar = 100 µm). (B) Fluorescence intensity profile along the yellow arrow region in different groups of tumor spheroids. (C) In vivo fluorescent images of MDA‐MB‐231 tumor‐bearing mice at different time points after i.v. injection of AMPM. (D) Mean fluorescence intensity in the tumor sites at different time points (ns = non‐significant). (E) Ex vivo major tissues (heart, liver, spleen, lung, kidney, and tumor) imaging at 12 and 48 h post‐injection. (F) The corresponding mean fluorescence intensity of organs in Figure 4E. (G) In vivo photothermal imaging of mice treated by PBS, AMP, and AMPM. The tumor was irradiated under 808 nm laser for 5 min with power density of 0.5 W•cm^−2^. (H) Temperature statistics at the tumor site according to (G).

The dense ECM of solid tumors impedes nanoparticle drug delivery. However, the photothermal effect produced by NIR irradiation offers distinct advantages in cancer treatment, as it can damage tumor cells and enhance the permeability of the ECM. Accordingly, the present study employed the photothermal effect of AMPM to simulate its penetration into tumors by constructing MCSs in vitro. The AMPM was loaded with Nile red. The fluorescence distribution of AMPM and AMPM+NIR was characterized using CLSM, indicated by red signals (Figure 5A). AMPM alone localized only to the MCS surface, with signals diminishing by 100 µm depth; AMPM+NIR enabled penetration into the MCS interior (∼100 µm) with sustained intensity, confirming enhanced permeability (Figure 5B). We quantified the penetration distance of AMPM into spheroids with/without NIR irradiation by measuring the distance from the spheroid edge to the deepest site where fluorescently labeled AMPM was detectable (Figure S28). The average penetration depth was 26.5 µm in non‐irradiated spheroids, while it significantly increased to 232.5 µm post‐NIR irradiation, providing evidence that NIR irradiation enhances AMPM penetration. Meanwhile, We performed lysosome co‐localization assays to characterize the internalization pathway and efficiency of AMPM (Figure S29). The confocal microscopy images show strong co‐localization between Nile red labeled AMPM and lysosomal markers at 2 h post‐incubation, indicating active cellular internalization.

Prior to in vivo tumor treatment, a mouse tumor model was established to assess the accumulation time of Nile Red‐labeled AMPM at the tumor site (Figure 5C,D). In following tail vein injection of AMPM, maximum accumulation at the tumor site was observed between 8 and 12 h. At 24 h, the fluorescence signal at the tumor site significantly decreased, and were nearly undetectable at 48 h. 24 and 48 h after the injection of AMPM, the mice were sacrificed, and fluorescence imaging was performed on the excised organs and tumors. The significant fluorescence signals were detected in the liver, lungs, and tumor sites at 24 h (Figure 5E). However, these fluorescence signals diminished considerably at 48 h. Quantitative fluorescence analysis, as presented in Figure 5F, indicated that AMPM predominantly accumulated in the liver, kidneys, and tumor sites, with substantial accumulation observed at 12 h that significantly decreased by 48 h.

This study employs the photothermal effect to enhance tumor penetration and facilitate the release of drugs and oxygen. The images were captured at various time points following the tail vein injection of AMPM in tumor‐bearing mice 8 h post‐injection, using an 808 nm laser at a power of 0.5 W•cm^−2^ (Figure 5G). Both AMP and AMPM exhibit a response to the NIR laser by generating a thermal effect, resulting in a gradual temperature increase over time. In the AMP and AMPM groups, temperatures of 45.3°C and 45.9°C were recorded at the tumor site after 5 min, respectively, exceeding the phase transition temperature of PCM, ensuring the release of MTZ and oxygen. To investigate the penetration depth of MTZ in tumor tissues, we used fluorescein isothiocyanate (FITC) as a surrogate to study its penetration behavior in tumor tissues (Figure S30). After NIR irradiation, FITC‐ encapsulated AMPM penetrated deeper into tumors compared with the non‐irradiated group, and obvious signals were detected in tumor tissues, providing evidence for the penetration of AMPM in tumors.

### 2.5 Synergistic Radiosensitization of AMPM In Vivo

**FIGURE 6:**
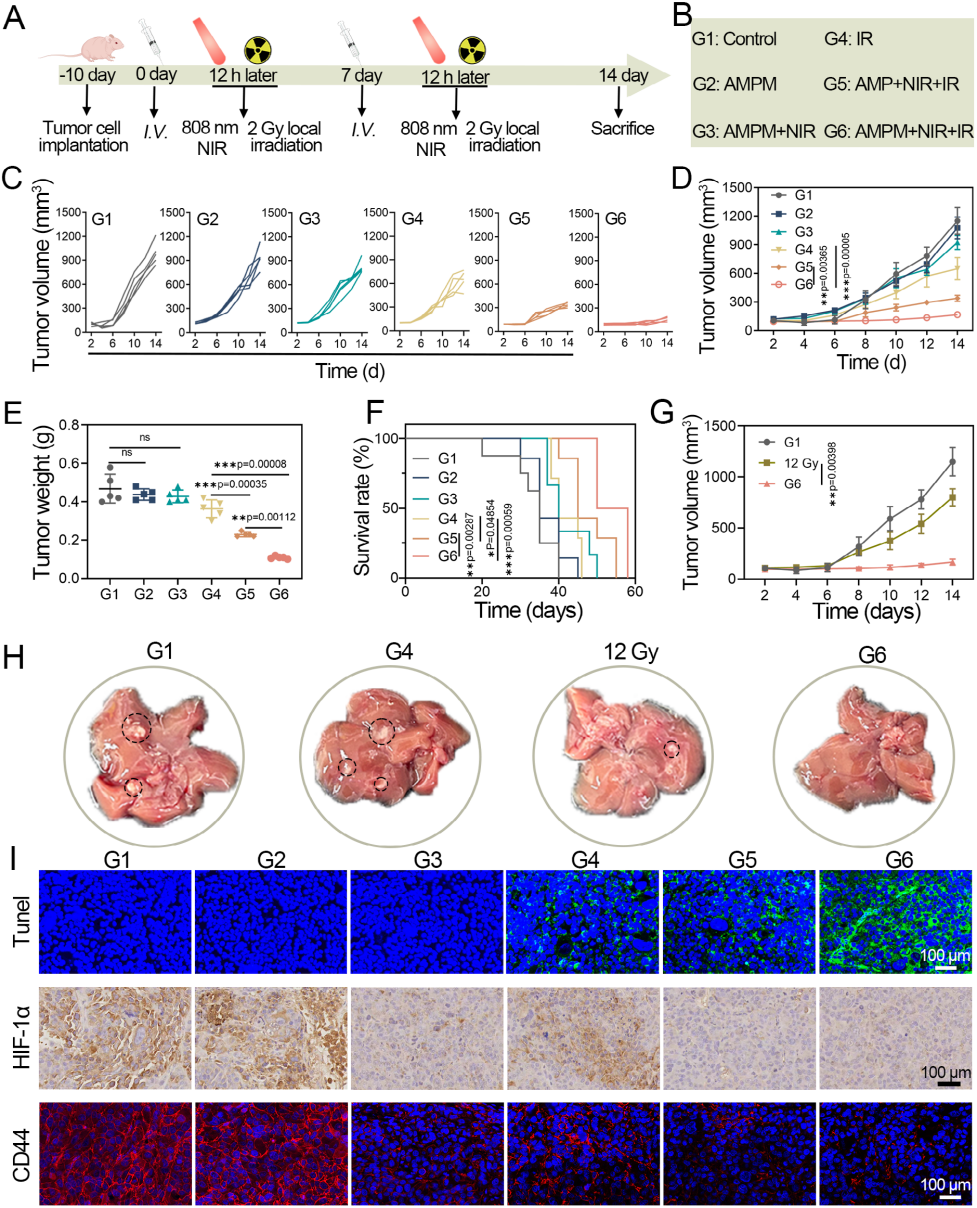
AMPM augments RT against MDA‐MB‐231 tumor‐bearing mice. (A) Schedule of tumor therapy. (B) Different treatment groups. (C) Individual tumor growth curves of the mice in the different groups. (D) Tumor volume curves for the various treatment groups (mean ± SD, *n* = 5). (E) Tumor weight of different groups (mean ± SD, ns = non‐significant). (F) Kaplan–Meier survival curves of each group (mean ± SD, *n* = 5). (G) Tumor volume curves for G1, 12 Gy, and G6 (mean ± SD, *n* = 5). (H) Representative images of the whole livers after different treatments. (I) TUNEL staining, immunohistochemistry images of HIF‐1α, and immunofluorescence images of CD44 at tumor sections after treatment by assigned formulations. ns = non‐significant. A *p* value of *p < 0.05, **p < 0.01, and ***p < 0.001 was considered statistically significant.

To verify whether the thermoresponsive delivery platform enhances RT for tumor growth suppression, its efficacy was evaluated in a breast cancer model. Tumor‐bearing BALB/c nude mice were intravenously administered PBS, AMP, or AMPM on days 0 and 7. At 8 h post‐injection, groups G3, G5, and G6 received 808 nm laser irradiation, followed by γ‐ray irradiation (2 Gy) for G4, G5, and G6 (Figure 6A). Tumor size measurements over 14 days showed no significant growth inhibition in G2 (AMPM alone) or G3 (AMPM + NIR) compared to the control (Figure 6C,D), indicating negligible tumor‐inhibitory effects of AMPM or NIR at the tested energy density. G4 (4 Gy RT alone) achieved 35.6% tumor suppression. G5 (AMP + NIR + RT) showed enhanced suppression (71%), due to NIR‐triggered oxygen release from AMP synergizing with RT. G6 (AMPM + NIR + RT) exhibited the strongest efficacy (91.2% suppression) via NIR‐induced co‐release of oxygen and MTZ to potentiate RT. Tumor weight analysis (Figure 6E) confirmed significant differences between G6 and G5.

The survival rates of mice in different groups were observed and recorded within 60 days of treatment. The 60‐day survival rate of mice in the G6 group was 48.6%, showing a significant difference compared to the G4 group (Figure 6F). We added an additional experimental group using PCM‐encapsulated PFP (designated as PP) as a control for antitumor therapy. As shown in Figure S31B,C, no significant tumor volume inhibition was observed in the G7 group. After sacrificing the mice, compared to the Control group, tumor weights of the PP group showed no significant difference (Figure S31D). Survival analysis of G7 mice revealed a mortality rate of 50% at 30 days, with no significant difference from the Control group (Figure S31E). Figure S32 displays ex vivo tumor images of the mice. To further investigate the effects of AMPM on radiosensitization, we set up a group of mice for high‐dose irradiation with RT alone. In a high‐dose RT control (12 Gy alone), tumor suppression was 52.1%, which was significantly lower than G6 (Figure 6G). The combination of AMPM with RT can achieve better therapeutic effects at lower irradiation doses, thereby strengthening the radiosensitizing capacity.

Liver metastasis was evaluated across groups (Figure 6H). G4 (4 Gy RT) showed more metastasis than G1 group, while high‐dose RT (12 Gy) reduced but did not eliminate it. Notably, G6 (AMPM + NIR + RT) exhibited no significant liver metastasis, likely due to NIR‐triggered co‐release of drugs and oxygen, which synergizes with RT to alleviate tumor hypoxia, modulate stem cell differentiation, and inhibit migration. Quantitative graph of the number of liver metastatic tumors in different treatment groups was shown in Figure S33. Immunohistochemical and immunofluorescence analyses revealed G6 tumors had the strongest apoptotic effects (Figure 6I). The tumor tissues were characterized for the hypoxia factor HIF‐1α and the breast cancer stem cell surface marker CD44. We further explored the inhibitory effect of oxygen on HIF‐1α by immunofluorescence and WB experiments (Figure S34A,B). For cells cultured under hypoxia, the fluorescence intensity of HIF‐1α was significantly reduced in cells treated with AMPM + NIR by comparison with the control group. We further examined protein level changes to explore the expression of HIF‐1α (Figure S34C). Western blot results demonstrated significant reductions in HIF‐1α levels in the AMPM + NIR group. CAIX is a protein specifically overexpressed on the surface of hypoxic tumor cells. To indirectly assess the oxygen levels in tumor tissues, we performed CAIX immunofluorescence staining on tumor tissues from different treatment groups. The results showed that the expression of CAIX was significantly downregulated in the AMPM and AMP groups after NIR treatment (Figure S35). These results confirm that NIR‐stimulated oxygen release from AMPM downregulates CAIX, HIF‐1α, and CD44, alleviating tumor hypoxia and reducing CSCs stemness. In addition, we detected the expression of key proteins in the ECM of tumor tissues (Figure S36). Under near‐infrared (NIR) irradiation, AMPM significantly reduced the deposition of collagen I and fibronectin, regulated the structure of the ECM, which may alleviate the barrier to drug penetration.

We used flow cytometry to quantitatively determine the population of CSCs in tumor tissues from different treatment groups (Figure S37). The results demonstrate that, compared with the control group, the G3 (AMPM + NIR), G5 (AMP + NIR + RT), and G6 (AMPM + NIR + RT) groups show a significant decrease in the proportion of CD44^+^/CD24^−^ CSCs. This study used nano‐delivery particles AMPM to precisely deliver oxygen and RT sensitizers to deep tumors, aiming to overcome RT resistance induced by CSCs. Composed of thermoresponsive ABM, oxygen‐saturated PFP, and MTZ, AMPM employs PCM as a temperature‐sensitive gatekeeper—triggering controlled release of oxygen and MTZ under NIR‐induced mild hyperthermia (40°C). This design enhances tumor ECM permeability, facilitating deep‐tumor delivery of oxygen and MTZ. Oxygen improves radiosensitivity by alleviating hypoxia and reducing CSCs stemness; MTZ further potentiates RT by inhibiting radiation‐induced DNA damage repair.

### 2.6 Biosafety Characterization of AMPM

**FIGURE 7:**
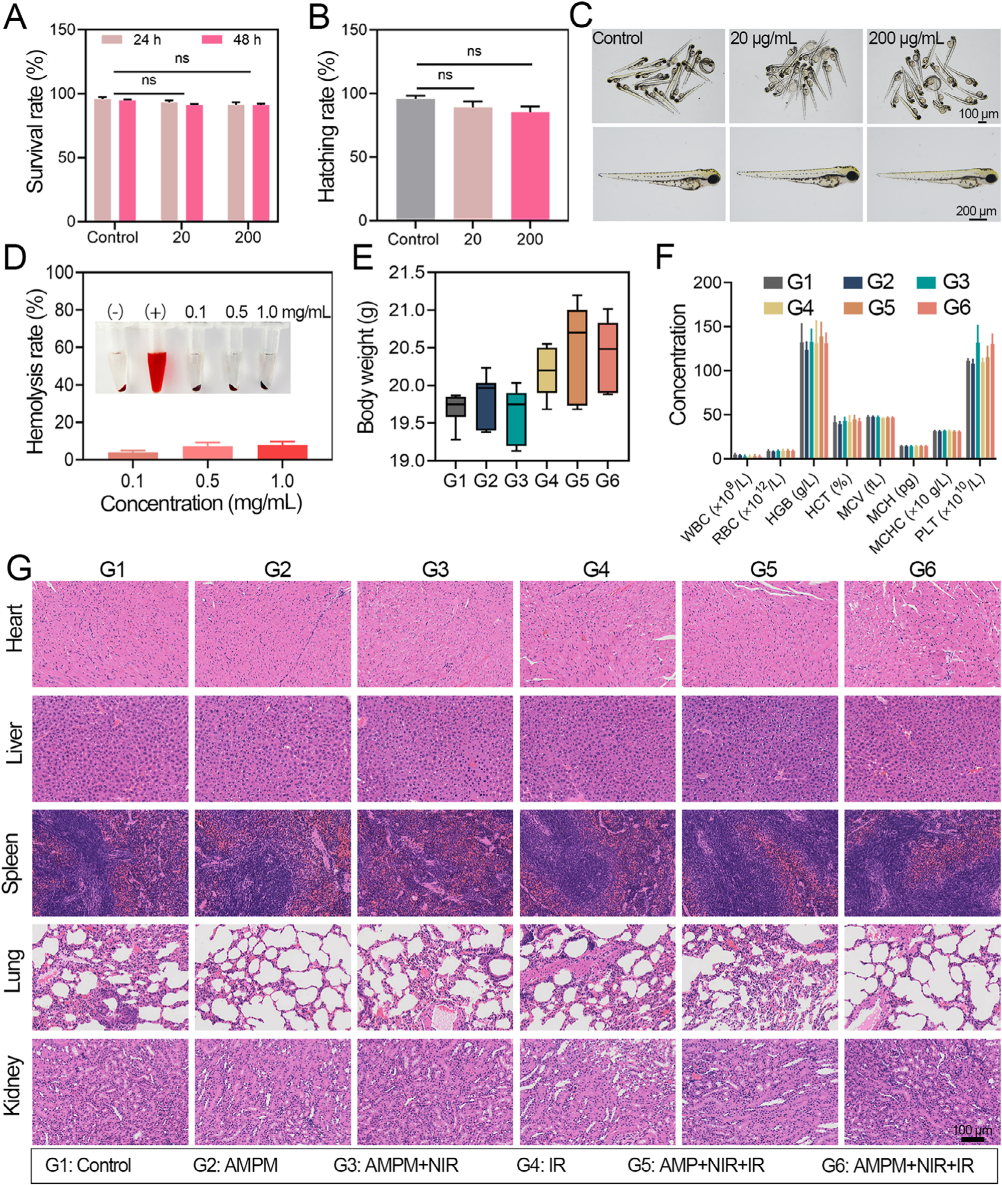
Biosafety evaluation of AMPM during treatment. (A) The survival rate of zebrafish incubated with different concentrations of AMPM (20 and 200 µg•mL^−1^) for 24 and 48 h. Hatching rate (B) and representative images (C) of zebrafish incubated with different concentrations of AMPM (20 and 200 µg•mL^−1^) after 72 h. (D) Hemolysis rates for different concentrations (0.1, 0.5, 1.0 mg•mL^−1^) of AMPM; inset: images of hemolysis. (E) Body weight changes of mice during the treatment of 21 days. Values are expressed as means ± SD (*N* = 5). (F) Hematological analysis results of mice after various treatments via intravenous injection. white blood cell (WBC), red blood cell (RBC), hemoglobin (HGB), hematocrit (HCT), mean corpuscular volume (MCV), mean corpuscular hemoglobin (MCH), mean corpuscular hemoglobin concentration (MCHC), platelet (PLT). (G) Representative photomicrographs of the heart, liver, spleen, lung, and kidney sections (H&E staining) of tumor‐bearing mice after the treatment of 14 days.

The biosafety of AMPM was systematically evaluated in vivo and in vitro. First, at the cellular level, the cytotoxicity of AMPM was investigated at various concentrations, and the cell viability was >85% even after 72 h of incubation, revealing the negligible cytotoxicity (Figure S38). Furthermore, as a model organism, zebrafish has advantages in safety evaluation due to its small body size, high reproductive rate, and short passage period. To evaluate the biosafety of AMPM, we incubated zebrafish embryos with different concentrations of AMPM to study the survival rate (Figure 7A) and hatching rate (Figure 7B) of the embryos. The results showed that 200 µg•mL^−1^ AMPM had no significant effect on the survival rate and hatching rate of zebrafish. Figure 7C shows representative pictures of zebrafish after different treatments. Further, we evaluated the hemolytic rate of AMPM, as shown in Figure 7D, compared to the control group, there was no significant difference observed for AMPM at a concentration of 1 mg•mL^−1^. After the treatment in vivo, there was no significant weight loss (Figure 7E) and no obvious abnormality in blood phase indicators (Figure 7F) observed. The H&E staining of the main organs (liver, spleen, kidneys, heart, and lungs) in mice was performed, and no obvious damage was observed (Figure 7G). Therefore, the strategy of AMPM responding to NIR to release oxygen and MTZ in combination with RT for achieving radiosensitization shows good biosafety.

## 3 Conclusions

In conclusion, the thermal responsive RT sensitization nano‐platform exhibits advantages in both performance and biocompatibility. By encapsulating the photothermal material ABM, the RT sensitizer, and oxygen‐pre‐saturated PFP within phase change materials, we have created a thermally responsive drug delivery platform. This therapeutic platform can effectively accumulate at the tumor site via the EPR effect, releasing the RT sensitizer MTZ and oxygen in response to thermal stimuli. Simultaneously, the thermal effect can enhance the permeability of the ECM in tumors, facilitating the deeper penetration of MTZ and oxygen. Oxygen can improve the hypoxic environment of tumors, reduce the expression of tumor stem cell CD44, and inhibit the expression of HIF‐1α. Compared to the control group, the inhibitory effect on tumor cell migration was increased by 87.6% in the wound healing assay. MTZ inhibits DNA damage repair caused by γ‐rays, achieving a radiosensitizing effect. In the mouse MDA‐MB‐231 tumor model, the AMPM treatment group exhibited a significant tumor suppressive effect, with a tumor suppression rate of 91.2%. The combined treatment of AMPM + NIR irradiation significantly reduces liver metastasis in a murine model of metastatic tumor compared to control groups. Combined treatment of AMPM irradiation significantly downregulate the expression of CSC stemness markers (CD44, Sox‐2, Nanog, and Otc‐4) in vitro spheroid models, and significantly reduced both CSCs numbers and expression of related genes. The nano‐platform can mitigate tumor resistance to RT, thereby introducing advanced treatment strategies for solid tumors. Although AMPM shows translational potential, several challenges must be addressed before clinical implementation. Regarding the 808 nm NIR light used in the platform, its limited tissue penetration depth may restrict application to superficial tumors or those accessible through specific interventions. In terms of formulation scale‐up and batch consistency for clinical‐grade AMPM production, large‐scale synthesis may face issues such as maintaining the stability of the nano‐platform structure and ensuring uniform drug loading. Microfluidic‐based scalable synthesis protocols can be developed to ensure the batch‐to‐batch reproducibility. In addition, extended pharmacokinetic, biodistribution, and long‐term safety studies are essential. While these preclinical studies are crucial, translating AMPM from the laboratory to the clinic will require rigorous validation across multiple dimensions.

## 4 Experimental Section

Experimental details are provided in Supporting Information.

## Author Contributions

Yuanfang Chen contributed to investigation, figure design, data curation, original draft writing, and conceptualization. Xueyin Hu and Ze Hu contributed to data curation and conceptualization. Changfen Bi, Yuwei Yang, and Guangyou Shi contributed to conceptualization. Lumeng Zhang contributed to data interpretation, manuscript refinement. Shuqin Li and Wenqing Xu contributed to conceptualization, review and editing. Luntao Liu contributed to conceptualization, validation, review and editing, and supervision. Yuanfang Chen, Xueyin Hu, and Ze Hu contributed equally. All authors have read and agreed to the published version of the manuscript.

## Conflicts of Interest

The authors declare no conflicts of interest.

## Supporting information




**Supporting File 1**: exp270126‐sup‐0001‐SuppMat.docx.

**Supporting File 2**: exp270126‐sup‐0002‐tableS1.docx.

## Data Availability

Data available on request from the authors: The data that support the findings of this study are available from the corresponding author upon reasonable request.

## References

[1] B. Jovanović , S. E. Church , K. M. Gorman , et al., “Integrative Multiomic Profiling of Triple‐Negative Breast Cancer for Identifying Suitable Therapies,” Clinical Cancer Research 30 (2024): 4768–4779, 10.1158/1078-0432.CCR-23-1242. 39136550 PMC11474168

[2] R. A. Leon‐Ferre and M. P. Goetz , “Advances in Systemic Therapies for Triple Negative Breast Cancer,” BMJ 381 (2023): e071674, 10.1136/bmj-2022-071674. 37253507

[3] S. Zhu , Y. Wu , B. Song , et al., “Recent Advances in Targeted Strategies for Triple‐Negative Breast Cancer,” Journal of Hematology & Oncology 16 (2023): 100, 10.1186/s13045-023-01497-3. 37641116 PMC10464091

[4] J. B. Schaubaecher , B. Smiljanov , F. Haring , et al., “Procoagulant Platelets Promote Immune Evasion in Triple‐Negative Breast Cancer,” Blood 144 (2024): 216–226, 10.1182/blood.2023022928. 38648571

[5] B. Liu , H. Zhou , L. Tan , K. T. H. Siu , and X.‐Y. Guan , “Exploring Treatment Options in Cancer: Tumor Treatment Strategies,” Signal Transduction and Targeted Therapy 9 (2024): 175, 10.1038/s41392-024-01856-7. 39013849 PMC11252281

[6] I. Dagogo‐Jack and A. T. Shaw , “Tumour Heterogeneity and Resistance to Cancer Therapies,” Nature Reviews Clinical oncology 15 (2018): 81–94, 10.1038/nrclinonc.2017.166. 29115304

[7] F. Yang , “The Integration of Radiotherapy With Systemic Therapy in Advanced Triple‐Negative Breast Cancer,” Critical Reviews in Oncology/Hematology 204 (2024): 104546. 39476993 10.1016/j.critrevonc.2024.104546

[8] G. Patel , A. Prince , and M. Harries , “Advanced Triple‐Negative Breast Cancer,” Seminars in Oncology Nursing 40 (2024): 151548. 38008654 10.1016/j.soncn.2023.151548

[9] C. Sousa , M. Cruz , A. Neto , et al., “Neoadjuvant Radiotherapy in the Approach of Locally Advanced Breast Cancer,” ESMO Open 5 (2020): e000640. 10.1136/esmoopen-2019-000640 PMC7082639 32152044

[10] M. Charpentier , S. Spada , S. J. Van Nest , and S. Demaria , “Radiation Therapy‐Induced Remodeling of the Tumor Immune Microenvironment,” Seminars in Cancer Biology 86 (2022): 737–747, 10.1016/j.semcancer.2022.04.003. 35405340

[11] T. Suwa , M. Kobayashi , J.‐M. Nam , and H. Harada , “Tumor Microenvironment and Radioresistance,” Experimental & Molecular Medicine 53 (2021): 1029–1035, 10.1038/s12276-021-00640-9. 34135469 PMC8257724

[12] Y. Wu , Y. Song , R. Wang , and T. Wang , “Molecular Mechanisms of Tumor Resistance to Radiotherapy,” Molecular Cancer 22 (2023): 96, 10.1186/s12943-023-01801-2. 37322433 PMC10268375

[13] G. Dontu , M. Al‐Hajj , W. M. Abdallah , M. F. Clarke , and M. S. Wicha , “Stem Cells in Normal Breast Development and Breast Cancer,” Cell Proliferation 36 (2003): 59–72, 10.1046/j.1365-2184.36.s.1.6.x. 14521516 PMC6495427

[14] F. R. Greten , “Tumour Stem‐Cell Surprises,” Nature 543 (2017): 626–627, 10.1038/543626a. 28358084

[15] M. Krause , A. Dubrovska , A. Linge , and M. Baumann , “Cancer Stem Cells: Radioresistance, Prediction of Radiotherapy Outcome and Specific Targets for Combined Treatments,” Advanced Drug Delivery Reviews 109 (2017): 63–73, 10.1016/j.addr.2016.02.002. 26877102

[16] C. Peitzsch , A. Tyutyunnykova , K. Pantel , and A. Dubrovska , “Cancer Stem Cells: The Root of Tumor Recurrence and Metastases,” Seminars in Cancer Biology 44 (2017): 10–24, 10.1016/j.semcancer.2017.02.011. 28257956

[17] X. Zhuo , R. Aishajiang , Y. Liang , et al., “Empowering Radiotherapy: Harnessing Nanomedicines to Enhance Radiation Response and Boost Antitumor Efficacy,” Coordination Chemistry Reviews 520 (2024): 216140, 10.1016/j.ccr.2024.216140.

[18] G. Li , D. Wang , Y. Zhai , et al., “Glycometabolic Reprogramming‐Induced XRCC1 Lactylation Confers Therapeutic Resistance in ALDH1A3‐overexpressing Glioblastoma,” Cell Metabolism 36 (2024): 1696–1710.e10, 10.1016/j.cmet.2024.07.011. 39111285

[19] D. C. Singleton , A. Macann , and W. R. Wilson , “Therapeutic Targeting of the Hypoxic Tumour Microenvironment,” Nature Reviews Clinical Oncology 18 (2021): 751–772, 10.1038/s41571-021-00539-4. 34326502

[20] I. Telarovic , R. H. Wenger , and M. Pruschy , “Interfering with Tumor Hypoxia for Radiotherapy Optimization,” Journal of Experimental & Clinical Cancer Research 40 (2021): 197, 10.1186/s13046-021-02000-x. 34154610 PMC8215813

[21] H. Dou , Z. Luo , H. Wang , et al., “Tumor Microenvironment‐Responsive Intelligent Nanoplatform With Oxygen Self‐Supply for Synergistic Chemotherapy/Photodynamic Therapy/Photothermal Therapy against Hypoxic Tumors,” Chemical Engineering Journal 487 (2024): 150523, 10.1016/j.cej.2024.150523.

[22] R. Kv , T.‐I. Liu , I. L. Lu , et al., “Tumor Microenvironment‐Responsive and Oxygen Self‐Sufficient Oil Droplet Nanoparticles for Enhanced Photothermal/Photodynamic Combination Therapy Against Hypoxic Tumors,” Journal of Controlled Release 328 (2020): 87–99, 10.1016/j.jconrel.2020.08.038. 32858076

[23] W. Wang , H. Zheng , J. Jiang , et al., “Engineering Micro Oxygen Factories to Slow Tumour Progression via Hyperoxic Microenvironments,” Nature Communications 13 (2022): 4495, 10.1038/s41467-022-32066-w. PMC9345862 35918337

[24] Q. Wang , H. Cao , X. Hou , et al., “Cancer Stem‐Like Cells‐Oriented Surface Self‐Assembly to Conquer Radioresistance,” Advanced Materials 35 (2023): 2302916, 10.1002/adma.202302916. 37288841

[25] A. Choudhury , M. A. Cady , C.‐H. G. Lucas , et al., “Perivascular NOTCH3+ Stem Cells Drive Meningioma Tumorigenesis and Resistance to Radiotherapy,” Cancer Discovery 14 (2024): 1823–1837. 38742767 10.1158/2159-8290.CD-23-1459 PMC11452293

[26] T. Sun , B. Liu , Y. Cao , Y. Li , L. Cai , and W. Yang , “AMPK‐Mediated CD47 H3K4 Methylation Promotes Phagocytosis Evasion of Glioma Stem Cells Post‐Radiotherapy,” Cancer Letters 583 (2024): 216605, 10.1016/j.canlet.2023.216605. 38218171

[27] B. C. Prager , Q. Xie , S. Bao , and J. N. Rich , “Cancer Stem Cells: The Architects of the Tumor Ecosystem,” Cell Stem Cell 24 (2019): 41–53, 10.1016/j.stem.2018.12.009. 30609398 PMC6350931

[28] M. Tutter , C. Schug , K. A. Schmohl , et al., “Regional Hyperthermia Enhances Mesenchymal Stem Cell Recruitment to Tumor Stroma: Implications for Mesenchymal Stem Cell‐Based Tumor Therapy,” Molecular Therapy 29 (2021): 788–803, 10.1016/j.ymthe.2020.10.009. 33068779 PMC7854278

[29] X. Wu , Y. Zhu , W. Huang , et al., “Hyperbaric Oxygen Potentiates Doxil Antitumor Efficacy by Promoting Tumor Penetration and Sensitizing Cancer Cells,” Advancement of Science 5 (2018): 1700859, 10.1002/advs.201700859. PMC6097095 30128223

[30] J. Gao , H. Qin , F. Wang , et al., “Hyperthermia‐Triggered Biomimetic Bubble Nanomachines,” Nature Communications 14 (2023): 4867, 10.1038/s41467-023-40474-9. PMC10421929 37567901

[31] S. He , X. Gou , S. Zhang , et al., “Nanodelivery Systems as a Novel Strategy to Overcome Treatment Failure of Cancer,” Small Methods 8 (2024): 2301127, 10.1002/smtd.202301127. 37849248

[32] Y. Xia , S. Fu , Q. Ma , Y. Liu , and N. Zhang , “Application of Nano‐Delivery Systems in Lymph Nodes for Tumor Immunotherapy,” Nano‐Micro Letters 15 (2023): 145, 10.1007/s40820-023-01125-2. 37269391 PMC10239433

[33] M. I. Priester and T. L. M. ten Hagen , “Image‐Guided Drug Delivery in Nanosystem‐Based Cancer Therapies,” Advanced Drug Delivery Reviews 192 (2023): 114621, 10.1016/j.addr.2022.114621. 36402247

[34] Z. Cao , J. Liu , and X. Yang , “Deformable Nanocarriers for Enhanced Drug Delivery and Cancer Therapy,” Exploration 4 (2024): 20230037, 10.1002/EXP.20230037. 39439489 PMC11491306

[35] A. D. Theocharis , S. S. Skandalis , C. Gialeli , and N. K. Karamanos , “Extracellular Matrix Structure,” Advanced Drug Delivery Reviews 97 (2016): 4–27. 26562801 10.1016/j.addr.2015.11.001

[36] Y. X. Zhu , H. R. Jia , Y. W. Jiang , et al., “A Red Blood Cell‐Derived Bionic Microrobot Capable of Hierarchically Adapting to Five Critical Stages in Systemic Drug Delivery,” Exploration 4 (2024): 20230105, 10.1002/EXP.20230105. 38855612 PMC11022606

[37] J. Insua‐Rodríguez and T. Oskarsson , “The Extracellular Matrix in Breast Cancer,” Advanced Drug Delivery Reviews 97 (2016): 41–55, 10.1016/j.addr.2015.12.017. 26743193

[38] M. Li , Y. Zhang , Q. Zhang , and J. Li , “Tumor Extracellular Matrix Modulating Strategies for Enhanced Antitumor Therapy of Nanomedicines,” Mater Today Bio 16 (2022): 100364, 10.1016/j.mtbio.2022.100364. PMC9305626 35875197

[39] Q. Zhou , S. Shao , J. Wang , et al., “Enzyme‐Activatable Polymer–Drug Conjugate Augments Tumour Penetration and Treatment Efficacy,” Nature Nanotechnology 14 (2019): 799–809, 10.1038/s41565-019-0485-z. 31263194

[40] Y. Shen , Y. Zou , B. Bie , and Y. Lv , “Hierarchically Released Liquid Metal Nanoparticles for Mild Photothermal Therapy/Chemotherapy of Breast Cancer Bone Metastases via Remodeling Tumor Stromal Microenvironment,” Advanced Healthcare Materials 12 (2023): 2301080, 10.1002/adhm.202301080. 37436138

[41] M. Zhan , X. Yu , W. Zhao , et al., “Extracellular Matrix‐Degrading STING Nanoagonists for Mild NIR‐II Photothermal‐Augmented Chemodynamic‐Immunotherapy,” Journal of Nanbiotechnology 20 (2022): 23, 10.1186/s12951-021-01226-3. PMC8740364 34991618

[42] L. P. Liew , A. Shome , W. W. Wong , et al., “Design, Synthesis and Anticancer Evaluation of Nitroimidazole Radiosensitisers,” Molecules (Basel, Switzerland) 28 (2023): 4457, 10.3390/molecules28114457. 37298933 PMC10254852

[43] C. Li , Y. Zhang , Z. Li , et al., “Light‐Responsive Biodegradable Nanorattles for Cancer Theranostics,” Advanced Materials 30 (2018): 1706150, 10.1002/adma.201706150. 29271515

[44] X. Wang , Y. Mao , C. Sun , Q. Zhao , Y. Gao , and S. Wang , “A Versatile Gas‐Generator Promoting Drug Release and Oxygen Replenishment for Amplifying Photodynamic‐Chemotherapy Synergetic Anti‐Tumor Effects,” Biomaterials 276 (2021): 120985, 10.1016/j.biomaterials.2021.120985. 34229242

[45] X. Liang , W. Chen , C. Wang , et al., “A Mesoporous Theranostic Platform for Ultrasound and Photoacoustic Dual Imaging‐Guided Photothermal and Enhanced Starvation Therapy for Cancer,” Acta Biomaterialia 183 (2024): 264–277, 10.1016/j.actbio.2024.05.040. 38815685

[46] M. W. Luczak and A. Zhitkovich , “Monoubiquitinated γ‐H2AX: Abundant Product and Specific Biomarker for Non‐Apoptotic DNA Double‐Strand Breaks,” Toxicology and Applied Pharmacology 355 (2018): 238–246, 10.1016/j.taap.2018.07.007. 30006243 PMC6754567

[47] V. Raavi , V. Perumal , and S. F. D. Paul , “Potential Application of γ‐H2AX as a Biodosimetry Tool for Radiation Triage,” Mutation Research Reviews in Mutation Research 787 (2021): 108350. 34083048 10.1016/j.mrrev.2020.108350

[48] A. Collins , P. Møller , G. Gajski , et al., “Measuring DNA Modifications With the Comet Assay: A Compendium of Protocols,” Nature Protocols 18 (2023): 929–989, 10.1038/s41596-022-00754-y. 36707722 PMC10281087

[49] E. Bivehed , B. Hellman , L. Wenson , B. Stenerlöw , O. Söderberg , and J. Heldin , “Visualizing DNA Single‐ and Double‐Strand Breaks in the Flash Comet Assay by DNA Polymerase‐Assisted End‐Labelling,” Nucleic Acids Research 52 (2024): e22, 10.1093/nar/gkae009. 38261985 PMC10899772

[50] S. Basu , Y. Dong , R. Kumar , C. Jeter , and D. G. Tang , “Slow‐Cycling (Dormant) Cancer Cells in Therapy Resistance, Cancer Relapse and Metastasis,” Seminars in Cancer Biology 78 (2022): 90–103, 10.1016/j.semcancer.2021.04.021. 33979674 PMC8576068

[51] L. Ma , B. Qiu , J. Zhang , et al., “Survival and Prognostic Factors of Non‐Small Cell Lung Cancer Patients with Postoperative Locoregional Recurrence Treated With Radical Radiotherapy,” Chinese Journal of Cancer 36 (2017): 93, 10.1186/s40880-017-0261-0. 29228994 PMC5725840

[52] M. Rafat , T. A. Aguilera , M. Vilalta , et al., “Macrophages Promote Circulating Tumor Cell–Mediated Local Recurrence Following Radiotherapy in Immunosuppressed Patients,” Cancer Research 78 (2018): 4241–4252, 10.1158/0008-5472.CAN-17-3623. 29880480 PMC6072588

[53] D. Bayik and J. D. Lathia , “Cancer Stem Cell‐Immune Cell Crosstalk in Tumour Progression,” Nature Reviews Cancer 21 (2021): 526–536. 34103704 10.1038/s41568-021-00366-w PMC8740903

[54] S. Shen , X. Xu , S. Lin , et al., “A Nanotherapeutic Strategy to Overcome Chemotherapeutic Resistance of Cancer Stem‐Like Cells,” Nature Nanotechnology 16 (2021): 104–113, 10.1038/s41565-020-00793-0. 33437035

[55] Y. Xiong , Z. Yong , C. Xu , et al., “Hyperbaric Oxygen Activates Enzyme‐Driven Cascade Reactions for Cooperative Cancer Therapy and Cancer Stem Cells Elimination,” Advancement of Science 10 (2023): 2301278, 10.1002/advs.202301278. PMC10375084 37114827

