# Supplementary material for: Phase‐Change Assembling Nanostructures Synergistically Potentiate Tumor Radiosensitivity by Reducing the Stemness of Cancer Stem‐Like Cells
Source: Exploration (Beijing). 2026 Feb 11;6(1):20250179. doi: 10.1002/EXP.20250179 (PMC12970158; doi:10.1002/EXP.20250179)
Supplement: Supplementary file 1 — Supporting File 1: exp270126‐sup‐0001‐SuppMat.docx. [file EXP2-6-20250179-s002.docx]

**Supporting Information**

**Supplementary experimental section:**

**1. General information**

**1.1 Materials**

The 4-Hydroxyacetophenone, 4-Hydroxybenzaldehyde, N,N-Dimethylformamide(DMF), Iodomethane, 3-Bromopropyne,Cuprousiodide, Diethylamine, Nitromethane, Ammonium acetate, Boron trifluoride diethyl etherate, diisopropylethylamine, potassium carbonate were purchased from Shanghai Aladdin with purity of 99 %. Reagents were purchased from Tianjin Jiangtian Chemical Technology Co.Ltd without further purification, unless otherwise noted. Anhydrous dichloromethane (DCM) was distilled over CaH_2_, and THF was distilled over sodium and stored under argon. Silica gel (300-400 mesh) was used for column chromatography. The phase-change material (PCM) used in this study is a binary mixture of lauric acid (LA, purity ≥ 99%, Aladdin) and stearic acid (SA, purity ≥ 98%, Aladdin) with a molar ratio of 4:1 (LA : SA). The cell counting kit-8 assay (CCK-8) was purchased from Beyotime Institute of Biothechnology. Fetal bovine serum (FBS), 3-Dulbecco’s Modified Eagle’s Medium (DMEM) medium, and trypsin were obtained from Invitrogen Corporation. PE-conjugated anti-human CD24 (PE-CD24, BD Pharmingen, 566593), FITC-conjugated anti-human CD44 (FITC-CD44, Biolegend, 332014), rabbit anti-human HIF-1α antibody (Invitrogen, Polyclonal, PA1184), Calcein AM cell-permeant dyeand and propidium iodide (PI) were acquired from Thermo Fisher Scientific (USA). All animal experiments were carried out in full compliance with the Animal Care and Use of Laboratory Animals of Peking Union Medical College. The animal ethics review number is IRM-DWLL-2023179. Female BALB/c nude mice (6-8 weeks, 17-20 g) were purchased from Vital River laboratory animal technology Co., Ltd. (Beijing, China) and acclimated to the environment (specific-pathogen-free, 22 ^o^C ± 2 ^o^C, 50 % humidity, and 12 h light/dark cycle) for seven days with free access to a standard diet.

**1.2 Characterization**

The ^1^H NMR and ^13^C NMR spectra were recorded on a Varian Inova 300 MHz NMR spectrometer using tetramethylsilane (TMS) as the internal standard. Multiplicities for proton signals are abbreviated as s, d, t, q, p, and m for singlet, doublet, triplet, quartet, pentet, and multiplet, respectively. UV/vis absorption spectra were recorded on an Agilent Technologies Cary 500 UV/vis spectrophotometer. The spectra were recorded in quartz glass cuvettes, and the extinction coefficient ε was calculated according to Lambert-Beer’s law. The fluorescence spectra were measured under ambient conditions on an Edinburgh FLS980 spectrofluorometer. TEM measurements were performed on a Hitachi HT7700 transmission electron microscope, operating at an acceleration voltage of 80 kV. The sample (100 μM) was drop cast on 400-mesh Formvar copper grids coated with carbon at room temperature for 20 min. Staining was performed by a drop of uranyl acetate aqueous solution (0.5 %) onto the copper grid. The samples were drop-cast on mica or HOPG (highly ordered pyrolytic graphite). DLS (Zetasizer Nano ZS) analyzer was used to measure the size of AMPM. The temperature was recorded by an infrared thermograph Thermal Imager of FLIR E64501.

**2. Experimental methods**

**2.1 Synthesis of AB**

AB were synthesized and characterized according to our previous work ^[1]^ (Scheme S1, Figure S1-8).

**SCHEME S1** Synthesis of AB. Reagents and conditions: (i) HC≡CCH_2_Br, K_2_CO_3_, DMF, N_2_ protection, r.t., 24 h, 90%; (ii) CH_3_I, K_2_CO_3_, DMF, N_2_ protection, 80 °C, reflux, 24 h, 92%; (iii)KOH, C₂H₅OH, H_2_O, r.t., 6 h; 97%; (iv) CH_3_NO_2_, (C_2_H_5_)_2_NH, CH_3_OH, r.t., reflux, 12 h, 70%; (v) CH_3_COONH_4_ ,130 °C, reflux, 12 h, 40%; (ⅵ) BF_3_·Et_2_O, (C_2_H_5_)_3_N, CH_2_Cl_2_, r.t., reflux, 56%; (ⅶ) CuI, CH_3_CN, H_2_O, THF, r.t., 24 h, 80%.

**Compound 8:** 4-Hydroxyacetophenone (5.45 g, 40 mmol) and K_2_CO_3_ (8.29 g, 60 mmol) were placed in a 500 mL two-neck round-bottom flask under N_2_ protection. DMF (30 mL) was added to the flask and stirred for one hour before adding 3-Bromopropyne (5.71 g, 48 mmol). The reaction mixture was stirred for 24 h at room temperature under N_2_ for 24 h. Then Ethyl Acetate (300 mL) and Ice water (100 mL) was added. The organic layer was separated, washed with water (3×100 mL) and dried over anhydrous magnesium sulfate. Further purification by column chromatography on silica eluting with CH_2_Cl_2_/hexane (1:2) and evaporation of the solvent gave the target compound as a White powder (6.3 g 90%).^1^H NMR: (300 MHz, CDCl_3_): δ 7.89 (d, *J* = 8.9 Hz, 1H), 6.96 (d, *J* = 8.9 Hz, 1H), 4.70 (d, *J* = 2.4 Hz, 1H), 2.64 – 2.44 (m, 2H).

**Compound 7**: 4-Hydroxybenzaldehyde (5.88 g, 40 mmol) and K_2_CO_3_ (22.00 g, 160 mmol) were placed in a 500 mL two-neck round-bottom flask under N_2_ protection. DMF (50 mL) was added to the flask, and the mixture was stirred at 60 °C for one hour. Then, 3-Bromopropyne (5.71 g, 48 mmol) was added, and the reaction was heated to 80 °C for 12 h.Then Ethyl Acetate (300 mL) and water (100 mL) was added. The organic layer was separated, washed with water (3×100 mL) and dried over anhydrous magnesium sulfate. Further purification by column chromatography on silica eluting with CH_2_Cl_2_/hexane (1 : 2) and evaporation of the solvent gave the target compound as a yellow oily liquid (5.12g 94%).1H NMR: (300 MHz, CDCl_3_): δ 9.84 (s, 1H), 7.89 – 7.68 (m, 2H), 7.08 – 6.84 (m, 2H), 3.80 (s, 3H).

**Compound 6:** Compound 8 (3.2 g, 19 mmol) dissolved in a solution of 50 mL ethanol was added to a 500 mL flask, along with a solution of KOH (8.04 g, 72 mmol) dissolved in 15 mL water. Then, a solution of compound 7 (2.5 g, 19 mmol) in ethanol (20 mL) was added dropwise to the flask. The reaction mixture was stirred for 6 h at room temperature. The solid was filtrated and carefully washed to neutral with cold water. The crude product was purified by recrystallization from ethanol (5.32 g, 96%).^1^H NMR: (300 MHz, CDCl_3_): δ 8.04 (d, *J* = 8.9 Hz, 1H), 7.78 (d, *J* = 15.6 Hz, 1H), 7.60 (d, *J* = 8.7 Hz, 1H), 7.40 (s, 1H), 7.06 (d, *J* = 8.9 Hz, 1H), 6.94 (d, *J* = 8.7 Hz, 1H), 4.78 (d, *J* = 2.4 Hz, 1H), 3.86 (s, 2H), 2.56 (t, *J* = 2.4 Hz, 1H).

**Compound 5:** Compound 6 (5.00 g, 17.10 mmol), nitromethane (5.22 g, 85.50 mmol), and diethylamine (6.25 g, 85.50 mmol) were dissolved in ethanol (200 mL) and added to a 500 mL flask. The reaction mixture was then heated under reflux for 12 h. After cooling at room temperature, the solvent was removed in vacuum and the oily residue obtained was dissolved in ethyl acetate and washed with water (3×50 mL). The combined organic layers were washed with brine, dried over anhydrous magnesium sulfate, and concentrated. Further purification by column chromatography on silica eluting with CH_2_Cl_2_/hexane (1 : 1) and evaporation of the solvent gave the target compound as a yellowish oily residue (3.60 g, 60%).^1^H NMR: (300 MHz, CDCl_3_): δ 7.96 – 7.83 (m, 1H), 7.27 – 7.13 (m, 1H), 7.06 – 6.96 (m, 1H), 6.91 – 6.79 (m, 1H), 4.89 – 4.72 (m, 2H), 4.71 – 4.52 (m, 1H), 4.26 – 4.05 (m, 1H), 3.78 (s, 2H), 3.48 – 3.23 (m, 1H), 2.58 (t, *J* = 2.4 Hz, 1H).

**Compound 4:** Compound 5 (2.12 g, 6 mmol) and ammonium acetate (21.48 g, 300 mmol) were added to a 500 mL flask. The reaction mixture was heated to 130 °C and refluxed for 12 hours. The reaction mixture in the round-bottomed flask was allowed to cool to room temperature. Then CH_2_Cl_2_ (500 mL) and water (50 mL) was added. The organic layer was separated. The combined organic layers were washed with brine, dried over anhydrous magnesium sulfate, and concentrated. Further purification by column chromatography on silica eluting with CH_2_Cl_2_/hexane (3:1) and evaporation of the solvent gave the target compound as a yellowish oily residue (950 mg, 25%).^1^H NMR: (300 MHz, CDCl_3_): δ 8.03 (d, *J* = 7.8 Hz, 1H), 7.90 (d, *J* = 8.8 Hz, 1H), 7.13 (d, *J* = 8.8 Hz, 1H), 7.05 (s, 1H), 6.97 (d, *J* = 8.3 Hz, 1H), 4.81 (s, 1H), 3.89 (s, 2H), 2.59 (s, 1H).

**Compound 3:** Compound 4 (200 mg, 0.32 mmol) was placed in a 200 mL two-neck round-bottom flask. Anhydrous CH_2_Cl_2_ (30 mL) was added to the flask, and the mixture was stirred until Compound 4 completely dissolved. Then, diisopropylethylamine (1 mL, 5.75 mmol) and boron trifluoride diethyl etherate (1 mL, 8.13 mmol) were added to the flask. The reaction mixture was stirred under N_2_ protection for 24 h. After the completion of the reaction, The mixture was washed with water (50 mL), and organic layer was dried over anhydrous magnesium sulfate and evaporated to dryness. Purification by column chromatography on silica eluting with CH_2_Cl_2_/hexane (2:1) and evaporation of the solvent gave the product (72 mg, 34%).^1^H NMR: (300 MHz, CDCl_3_): δ 8.06 (dd, *J* = 8.6, 6.0 Hz, 1H), 7.08 (d, *J* = 9.0 Hz, 1H), 6.99 (d, *J* = 8.9 Hz, 1H), 6.93 (s, 1H), 4.76 (d, *J* = 2.4 Hz, 1H), 3.90 (s, 1H), 2.57 (t, *J* = 2.4 Hz, 1H).

**Compound AB**: Compound 3 (67 mg, 0.1 mmol) and CuI (76.2 mg, 0.4 mmol) were dissolved in a mixture of CH_3_CN: H_2_O: THF (15: 1: 15). Then, compound 2^[1]^ (43.5 mg, 0.23 mmol) was added. The reaction mixture was stirred vigorously at 55 ^o^C for 24 h, then the solvent was removed by vacuum rotary evaporation. The crude material was purified by column chromatography using MeOH/CH_2_Cl_2_ (1: 10) as eluent to yield AB as a dark blue solid (90 mg, 87%). ^1^H NMR: (300 MHz, CDCl_3_): δ 8.05 (dd, J = 8.0, 6.0 Hz, 1H), 7.91 (s, 1H), 7.10 (d, J = 8.4 Hz, 1H), 6.98 (d, J = 8.8 Hz, 1H), 6.92 (s, 1H), 5.29 (s, 1H), 4.57 (s, 1H), 3.89 (s, 1H), 3.61 (d, J = 5.5 Hz, 3H), 3.34 (s, 1H). ^13^C NMR: (75 MHz, CDCl_3_) δ =161.03, 160.52, 157.89, 145.56, 143.02, 142.97, 132.00, 131.07, 125.76, 125.08, 117.34, 115.51, 114.34, 72.34, 70.91, 70.85, 70.77, 69.45, 69.41, 59.39, 59.32, 55.86.

**2.2 Preparation of AMPM and AMP**

Materials and Preparations: Amphiphilic AB was dissolved in methanol (HPLC grade) at a concentration of 2 mg⋅mL^-1^, and then was stored at 10 ^o^C for 4 h to form a homogeneous ABM solution, ensuring complete dissolution. The ABM solution was centrifuged, and then ABM was dried in vacuum at room temperature for 3 days. Metronidazole (MTZ, purity ≥ 98%) was dissolved in PBS at 1mM as stock solution. Perfluoropentane (PFP, purity ≥ 99%) was in the oxygen flow for 30 min. Phase-change material (PCM, Lauric acid: Stearic acid = 4: 1 with melting point 40 ^o^C) was dissolved in ethanol at 10 mg⋅mL^-1^ and filtered through a 0.22 μm membrane to remove impurities.

Synthesis of AMPM and AMP: ABM (10 mg) and metronidazole (100 µL of 1mM PBS solution) was transferred into a two-neck flask with a rubber stopper. Then, the air inside the flask was evacuated for 1 min, and 100 µL of PFP was injected into the flask. The entire system was subjected to ultrasonic oscillation for 2 min at a temperature of 4 ^o^C. Then, 1 mL PCM solution (10 mg⋅mL^-1^) was added into the flask under continuous sonication at 4 ^o^C for 2 min and rapidly was cooled in ice bath. Then the mixture was centrifuged (1000 rpm⋅min^-1^, 5 min) and dried at room temperature to obtain AMPM. AMP was synthesized using the identical protocol as AMPM, excluding the addition of MTZ. This ensured that any differences in physicochemical properties between AMPM and AMP could be attributed specifically to MTZ loading.

**2.3 Particle size measurements**

DLS (Zetasizer Nano ZS) analyzer was used to measure the size of AMPM in PBS and 10 % FBS at different time points (24 h, 48 h and 72 h). The AMPM (50 μg⋅mL^-1^) solution was placed in a 1.0 mL plastic test tube, and the particle size changes at different time points were detected. For the AMPM post irradiation (50 μg⋅mL^-1^, under 808 nm of 0.5 W⋅cm^-2^ for 5 min), 1 mL of the irradiated solution was transferred into a plastic test tube, and its hydrodynamic size was measured.

**2.4 Preparation of the nile red loaded AMPM**

A total of 10 μL of Nile red methanol solution (1 mg⋅mL^-1^) was added into AMPM (1.0 mg⋅mL^-1^, 1 mL), and the mixture was shaken overnight. Then the solution was placed in asemipermeable membrane (MWCO: 2000 Da) and dialyzed indeionized water for 24 h.

**2.5 Photothermal measurement**

Photothermal heating curves of various concentrations of AMPM (25, 50, 75, and 100 μg⋅mL^-1^) were measured under 808 nm laser irradiation at different of power density (0.1, 0.2, 0.4, 0.5 W⋅cm^-2^). The temperature was recorded by an infrared thermograph Thermal Imager of FLIR E64501.

**2.6 *In vitro* heat and NIR laser induced bubble generation**

The AMPM solution is stored in a glass vial and immersed in a constant temperature water bath at 40 ^o^C for 2 min to observe the generation of heat-induced bubbles. Drop 20 μL of the sample onto a slide, cover it with a cover slip, and after irradiating with 808 nm laser at 0.5 W⋅cm^-2^ for 5 min, capture the exposed area using an optical microscope.

**2.7 Release behavior of metronidazole**

Place the AMPM PBS solution (1.0 mg⋅mL^-1^) in a dialysis tube (MWCO: 2000 Da) and immerse it in deionized water at different temperatures (room temperature, 37 ^o^C and 40 ^o^C). At various time points (30 min, 1 h, 2 h, 4 h, 8 h and 12 h), samples of the dialysis fluid was tested UV-visible spectra and calculated the concentration of metronidazole in the dialysis fluid based on the standard curve of metronidazole.

**2.8 Cell culture and sorting**

L929, MDA-MB-231 cells were kept in our lab all the time. All the cells were cultured in Dulbecco’s Modified Eagle Medium (DMEM) supplemented with 10 % (v / v) fetal bovine serum and 1 % penicillin-streptomycin. The portable multi-functional cell hypoxia culture chamber (MIC-101) is placed in the Thermo Scientific incubator for culturing cells under hypoxic conditions at 37 ^o^C under 1 % O_2,_ 5 % CO_2_ and 95 % relative humidity.

CSCs were collected by flow cytometry (FCM) using the Hoechst side population (SP) method, referring to a previous report. ^[2]^ Briefly, a cell suspension of adherent cells (1 × 10^6^ cells⋅mL^-1^) was preheated at 37 ^o^C for 5 min and then treated with Hoechst 33342 at a final concentration of 6 μg⋅mL^-1^ for 90 min. In the control reaction, verapamil hydrochloride (Sigma) was added to a final concentration of 100 μM. After incubation, cells were resuspended in medium at a concentration of 1 × 10^7^ cells⋅mL^-1^. Cells were stained with propidium iodide (PI) (1 μg⋅mL^-1^) for 10 min to assess cell viability and analyzed and sorted using a flow cytometer (Tune NxT, Thermo Scientific). The sorted cells were cultured in DMEM/F12 medium containing B27 (1×), epidermal growth factor (20 ng⋅mL^-1^), basic fibroblast growth factor (20 ng⋅mL^-1^), insulin (5 μg⋅mL^-1^), and penicillin (1%, v / v) in an ultralow attachment culture dish (Corning).

**2.9 Identification of stemness-related property**

Logarithmic-phase MDA-MB-231 parental cells and third-generation suspension CSCs after various treatments were prepared into a single-cell suspension, collecting cells at a density of 1 × 10^6^ cells⋅mL^-1^ in 1 mL within a 1.5 mL centrifuge tube. After centrifugation and washing three times, the cells were resuspended in 100 μL PBS. 5 μL CD24-PE and CD44-FITC antibodies were added separately, and then stored in the dark at 4 ^o^C for 30 min. After the incubation, the cells were washed three times with PBS, and 10 μL were dropped onto a slide, covered with a glass coverslip, and observed under a fluorescence microscope for antibody expression. Following the same procedure, 100 μL of the cell suspension were analyzed by flow cytometry within 1 hour.

To detect the CD44 expression at the gene level, the total RNA from the cells was extracted using a 200 μL TRIzol reagent (Takara). Under the instructions of the manufacturer, reverse transcription (SimpliAmp, USA) and real-time PCR (Bio-rad CFX Connect, USA) were performed sequentially. The list of used primers is shown in Table S1.

**2.10 Cytotoxicity evaluation**

To assess the *in vitro* cytotoxicity of the AMPM, L929 cells were seeded on 96-well plates and treated with various concentrations of AMPM (25, 50, 100, 200, and 500 μg⋅mL^-1^) for different durations (24, 36, and 72 h) at 37 ^o^C under 5 % CO_2_. Subsequently, 20 μL of CCK-8 solution was added to each well and incubated for 4 h. Cell viability was evaluated by measuring the absorbance of the cells at 419 nm using a microplate reader. Control experiments were performed by adding an equal volume of PBS, and all treatments were conducted under identical conditions. The cell viability rate was calculated using the following equation:

Cell viability = (OD_samples_ - OD_blank_) / (OD_Control_ - OD_blank_)) × 100 %

**2.11 MCSs (multicellular spheroids) model**

The MCSs of MDA-MB-231 and SP-MDA-MB-231 cells were fabricated to research the penetrating ability of AMPM *in vitro*. Agarose gel solution was added into serum-free DMEM (2 %, w / v) and heated to 80 ^o^C, then was coated a 96-well plate to prevented surface adsorption. MDA-MB-231 and SP-MDA-MB-231 cells (1000 cells per well) suspended in DMEM were seeded in the 96-well plate and cultured in a humidified atmosphere containing 5 % CO_2_ at 37 ^o^C for 1 week to form the MCSs model.

**2.12 low-cell-number tumorigenicity assays**

The sorted CD44⁺/CD24⁻ cells were inoculated at a density of 1×10³ cells into both the dorsal subcutaneous and axillary regions of nude mice. After inoculation, tumor volumes were measured regularly over a 2 - 4 week monitoring period, with tumor formation defined as a volume ≥50 mm³.

**2.13 Treatment parameters of G1-G6 *in vitro***

We divided different treatments into groups, with the grouping information as follows: G1 (normal saline, no laser irradiation); G2 (AMPM, dose of 100 mg⋅mL^-1^, no laser irradiation); G3 (AMPM, dose of 100 mg⋅mL^-1^, with laser irradiation with a power density of 0.5 W⋅cm^-^² for 5 min); G4 ( γ-ray, 4 Gy); G5 (AMP, dose of 100 mg⋅mL^-1^; 808 nm laser irradiation with a power density of 0.5 W⋅cm^-^² for 5 min; radiation therapy with a γ-ray dose of 4 Gy); G6 (AMPM, dose of 100 mg⋅mL^-1^; 808 nm laser irradiation with a power density of 0.5 W⋅cm^-^² for 5 min; radiation therapy with a γ-ray dose of 4 Gy).

**2.14 ROS detection**

The MDA-MB-231 cells were planted in 6-well plates at a density of 2.0 × 10^5^ cells⋅well^-1^ and cultured under hypoxia for 24 h. After attachment, the cells were incubated with AMP and AMPM (100 μg⋅mL^-1^) under 808 nm irradiation (0.5 W⋅cm^-2^) for 5 min, after 4 h incubation, the cells were irradiated with γ-ray irradiator (0.99 Gy min^-1^, Canada Gammacell-40) at dose of 4 Gy. Then, the cells were washed with PBS three times, and 1 mL of 5 µM DCFH-DA was added at 37 ^o^C for 20 min. After treatment, the fluorescence intensity was measured through Fluorescence microscopy and flow cytometry.

**2.15 Calcein-AM/PI staining**

The MDA-MB-231 cells were seeded in 6-well plates at a density of 1 × 10^5^ cells per well, co-cultured with the AMP and AMPM after adherence under hypoxia. The cells were irradiated under 808 nm laser (0.5 W⋅cm^-2^) for 5 min, after 4 h incubation, the cells were irradiated with γ-ray irradiator (0.88 Gy min^-1^, Canada Gammacell-40) at dose of 4 Gy. The cells were washed by PBS and stained by Calcein-AM (1 mM) and PI (0.1 mM) for 30 min, then observed through fluorescence microscope. After the same treatment as above, the MCSs of MDA-MB-231 with different formulations, they were simultaneously stained with Calcein-AM (1 mM) and PI (0.1 mM) for 10 min. Then the tumor spheroids were washed with 1 × assay buffer and fixed with 4 % paraformaldehyde for 10 min. After washing by 1 × assay buffer again, the tumor spheroids were observed by the fluorescence microscope.

**2.16 Apoptosis assays**

According to the instructions of the Annexin V-FITC apoptosis detection kit (Solarbio, CA1020, Beijing, China), an apoptosis detection experiment was conducted. In brief, MDA-MB-231 cells were seeded at a density of 1.0 × 10^5^ cells⋅well^-1^ in a 6-well plate. After attachment, the cells were mionectic co-incubated with the AMP and AMPM, and were irradiated under 808 nm laser (0.5 W⋅cm^-2^) for 5 min, after 4 h incubation, the cells were irradiated with γ-ray irradiator (0.88 Gy⋅min^-1^) at dose of 4 Gy. For the IR group, 4Gy of irradiation was performed after cell adherence. For the AMPM + NIR and AMPM groups, after co-incubation with AMPM after cell attachment, the cells were irradiated with and without NIR laser irradiation, respectively. Subsequently, the cells were collected for FITC and PI staining. Data analysis was performed using FlowJo VX software (Becton-Dickinson, New Jersey, USA).

**2.17 Colocalization analysis in cells**

1 mL of MDA-MB-231 suspension with a cell density of 2 × 10^4^ cells⋅well^-1^ was added in a confocal dish for incubation of 24 h. The Nile Red-labeled AMPM (100 μg⋅mL^-1^) were added to 1 mL of DMEM cultured MDA-MB-231 cells at 37 ^o^C for 2 h, and then Lyso-Tracker Green DND-26 (500 nM) was added to incubate with cells for 30 min. Finally, the confocal dish was observed under a 63 × oil microscope in a confocal microscope (Nikon, Eclipse Ti2, Tokyo, Japan).

**2.18 The scratch assay**

The scratch assay was employed to assess the inhibitory effects of different formulations on tumor cell invasion and metastasis. In 6-well plate, 7 × 10^5^ cells were seeded per well and incubated overnight to allow for adherence. Following attachment, the cells were incubated under hypoxic conditions with the AMP (100 μg⋅mL^-1^) and AMPM (100 μg⋅mL^-1^), and were irradiated under 808 nm laser (0.5 W⋅cm^-2^) for 5 min, after 4 h incubation, the cells were irradiated with γ-ray irradiator (0.88 Gy min^-1^) at dose of 4 Gy. For the IR group, 4 Gy of irradiation was performed after cell adherence. For the AMPM + NIR and AMPM groups, after co-incubation with AMPM after cell attachment, the cells were irradiated with and without NIR laser irradiation, respectively. After various treatments, floating cells were removed by washing with PBS three times, the wells were filled with culture medium containing 1 % serum and incubated at 37 ^o^C in a 5 % CO_2_ atmosphere. Samples were collected at 0 h and 72 h, and images were captured and documented using an inverted fluorescence microscope.

**2.19 Alkaline comet assay**

The alkaline single-cell gel electrophoresis assay, also known as the comet assay, was employed to investigate DNA damage. In brief, MDA-MB-231 cells were seeded at a density of 1.0 × 10^5^ cells per well in 6-well plates. Following attachment, the cells were incubated under hypoxic conditions with the AMP (100 μg⋅mL^-1^) and AMPM (100 μg⋅mL^-1^), and were irradiated under 808 nm laser (0.5 W⋅cm^-2^) for 5 min, after 4 h incubation, the cells were irradiated with γ-ray irradiator (0.88 Gy min^-1^) at dose of 4 Gy. For the IR group, 4 Gy of irradiation was performed after cell adherence. For the AMPM + NIR and AMPM groups, after co-incubation with AMPM after cell attachment, the cells were irradiated with and without NIR laser irradiation, respectively. Subsequently, the cells were harvested and suspended in PBS. A 100 µL volume of agarose gel with a standard melting point was evenly applied onto CometSlide™ slides, which were then refrigerated at 4 ^o^C for 10 min. A mixture comprising 10 µL of cell suspension and 75 µL of low melting point agarose was thoroughly combined and subsequently dispensed onto the slides. The slides were cooled at 4 ^o^C for 10 min to allow for solidification. Subsequently, 75 µL of low melting point agarose was dispensed onto the slides, covered with a cover glass, and left to solidify for 20 min at 4 ^o^C. Following solidification, the slides were immersed in pre-cooled lysis buffer for 2 h at 4 ^o^C. They were then transferred to a freshly prepared alkaline lysis solution (1 mmol⋅L^-1^ EDTA, 300 mmol⋅L^-1^ NaOH, in H_2_O) and incubated for 60 min. Afterward, the slides were stored in an alkaline electrophoresis solution and electrophoresis was carried out at 30 V for 30 min. Following electrophoresis, the slides were neutralized using 0.4 mM Tris-HCl buffer (pH = 7.5). Next, 20 µL of PI solution was applied to the slides for 10 min to enable dark staining. Finally, the slides were examined under a fluorescence microscope, and the Comet Assay Software Project (CASP 1.2.3, Trevigen, Maryland, USA) was employed to assess DNA damage.

**2.20 Detection of γ-H2AX**

The investigation of DNA double-strand breaks (DSBs) was conducted utilizing histone H2AX (γ-H2AX) as a marker. MDA-MB-231 cells were cultured at a density of 10 × 10^5^ cells per dish for confocal microscopy. After attachment, the cells and MCSs were co-cultured with the AMP (100 μg⋅mL^-1^) and AMPM (100 μg⋅mL^-1^) under hypoxic conditions. The cells and MCSs were irradiated under 808 nm laser (0.5 W⋅cm^-2^) for 5 min, after 4 h incubation, the cells and MCSs were irradiated with γ-ray irradiator (0.88 Gy⋅min^-1^, Canada Gammacell-40) at dose of 4 Gy. Subsequently, the cells were fixed with 4 % paraformaldehyde for 20 min, and then washed with PBS. They were then treated with 0.2 % Triton-X 100 for 15 min. Following another PBS wash, the cells were incubated overnight at 4 ^o^C with a rabbit polyclonal primary antibody against γ-H2AX (dilution 1: 1000; catalog No. ab2893; Abcam, Cambridge, MA, USA). After removing the primary antibody, the cells were washed three times with PBS and then incubated with a secondary antibody, goat anti-rabbit (diluted 1: 2000; cat. No. ab6939; Abcam), at room temperature for 1 h. To visualize the nuclei, DAPI staining was performed (cat. No. C0065, Solarbio, Beijing, China). Cell images were captured using a confocal microscope equipped with a 63× oil objective lens (Nikon, Eclipse Ti2, Tokyo, Japan). The foci in each image were analyzed using Image Pro Plus 8.0 software (Media Cybernetics, Maryland, USA).

**2.21 Determination of cellular protein expression by western blot.**

Cells were seeded in 6-well plates at a density of 5 × 10⁵ cells per well and cultured for 24 h in hypoxic incubator. After attachment, the cells were incubated with AMPM (50, 100 μg⋅mL^-1^) under 808 nm irradiation (0.5 W⋅cm^-2^) for 5 min, and the cells were further cultured for 12 h under hypoxic conditions. After washing the cells three times with PBS, the cells were lysed using RIPA Lysis Buffe supplemented with a protease inhibitor. The lysed samples were centrifuged at a speed of 12,000 rpm, and the supernatant was taken for quantitative detection using the BCA Protein Assay Kit. According to the BCA results, the lysate was diluted with RIPA Lysis Buffer to equalize the amount of total protein for each group, and then analyzed by Western Blot.

**2.22 Quantitative real-time PCR analysis**

Total RNA was isolated from cells, zebrafish embryos and murine brain tissues via TRIzol® Reagent (Takara), followed by DNase I treatment to eliminate genomic DNA contamination. The RNA purity and concentration were verified via a NanoDrop 2000 spectrophotometer (Thermo). Reverse transcription was performed with 1 μg of RNA via PrimeScript™ RT Master Mix (Yeason) under optimized thermal conditions (37 °C for 15 min; 85 °C for 5 s inactivation).

Following irradiation/drug treatment, the cells were washed twice with ice-cold PBS. Tumor tissues were collected from tumor-bearing mice on the 14th day after treatment. Then the tumors were cut into small pieces and digested with 1 mg⋅mL^-1^ collagenase Type II solution (Invitrogen, USA) for 2 h at 37 ^o^C. Afterward, the solution was filtered by a 70 μm membrane filter to obtain the single-cell suspension. Red blood cell lysis buffer was added into the above solution to remove the red blood cells for 2 min. Cytosolic fractions were isolated via gently pipetting freshly prepared cytosolic extraction buffer (10 mM Tris-HCl pH 7.4, 10 mM NaCl, 3 mM MgCl₂, 0.1% Triton X-100). After 5 min of incubation on ice, the samples were centrifuged at 12000 rpm for 30 min at 4°C. The supernatant (cytosolic fraction) was transferred to new tubes, and the pellets (nuclear fraction) were discarded. Cytosolic DNA was extracted via a FastPure Cell DNA isolation kit (Vazyme). Quality control was performed by quantifying nuclear gene GAPDH contamination. Cytosolic fractions with a GAPDH Ct > 32 cycles were considered free of significant nuclear DNA contamination. The mtDNA copy number was normalized to that of 18S rDNA as an endogenous control.

The qPCRs (20 μL final volume) included SYBR Green® Premix Ex Taq II (Roche), 10 μM gene-specific primers (sequences listed in Supplementary Table 1), and diluted cDNA (1: 10). The amplification parameters included initial denaturation (95 ^o^C, 30 s), 40 cycles of two-step amplification (95 ^o^C for 5 s, 60 ^o^C for 30 s), and melt curve analysis (60-95 ^o^C, 0.5 ^o^C⋅s^-1^ increments). All reactions were performed in triplicate with no-template controls. Relative gene expression was calculated via the ΔΔCt method and normalized to that of β-actin or rsp18. Statistical significance was determined via one-way ANOVA with Tukey's post hoc test in GraphPad Prism 9.0.

**2.23 *In vivo* fluorescence imaging**

For *in vivo* luminescence imaging, MDA-MB-231 tumor-bearing mice were intravenously injected with AMPM. The luminescence signals of the mice were collected (λ_ex_ = 528 nm) through an *in vivo* imaging system (In-Vivo Master, China). Images were taken at 4, 8, 12, 24, and 48 h postinjection. After 24 h and 48 h, the tumor and major organs were collected and the fluorescence intensity was analyzed to confirm the accurate therapeutic time.

**2.24 *In vivo* thermal imaging**

MDA-MB-231 tumor-bearing mice were intravenously injected with AMPM, AMP and PBS. Subsequently, the tumor was exposed to 808 nm laser for 5 min with a power density of 0.5 W⋅cm^-2^ after 12 h post-injection. The temperature of the tumor was monitored using an infrared thermograph, specifically the FLIR E64501 Thermal Imager.

**2.25 *In vivo* therapeutic study**

All animal experiments were carried out in full compliance with the Animal Care and Use of Laboratory Animals of Peking Union Medical College. The animal ethics review number is IRM-DWLL-2023179. Female BALB/c nude mice were utilized to establish MDA-MB-231 xenograft models following the aforementioned protocol. Once the tumor volume reached 50-70 mm^3^, the mice were randomly allocated into 6 groups (n = 5) including Control (G1, normal saline, tail vein injection), AMPM (G2, AMPM with tail vein injection, dose of 1 mg⋅kg^-1^ , no laser irradiation), AMPM + NIR (G3, AMPM with tail vein injection, dose of 1 mg⋅kg^-1^, with laser irradiation with a power density of 0.5 W⋅cm^-^² for 5 min), IR (G4, radiation therapy group with γ-ray of 4 Gy), AMP + NIR + IR (G5, AMP with tail vein injection, dose of 1 mg⋅kg^-1^; 808 nm laser irradiation with a power density of 0.5 W⋅cm^-^² for 5 min; radiation therapy with a γ-ray dose of 4 Gy), AMPM + NIR + IR (G6, AMPM with tail vein injection, dose of 1 mg⋅kg^-1^; 808 nm laser irradiation with a power density of 0.5 W⋅cm^-^² for 5 min; radiation therapy with a γ-ray dose of 4 Gy). The mice intravenously administered PBS, AMP and AMPM. After 12 h post-injection, all groups were exposed to 5 min of irradiation using 808 nm NIR laser with a power density of 0.5 W⋅cm^-2^. After 1 h of laser irradiation, the mice were exposed to local irradiation with 4 Gy of γ-ray. Tumor volume and body weights were recorded every other day over a period of 3 weeks. The lengths and widths of the tumors were measured using a digital caliper, and the tumor volume was calculated by the formula: V = length × width^2^ /2. Body weights were denoted as W. The presented data are expressed as mean ± SD (N = 5). After the mice were sacrificed through cervical vertebra dislocation, the main organs (heart, liver, spleen, lung, and kidney) and tumors were collected. The main organs would be used for biochemical and cytological analysis.

**2.26 Flow cytometry for evaluating CSC**

On day 14 after treatment, the mice were euthanized, and tumor tissues from one mouse in each group were collected. The tumor tissue was enzymatically digested with DNase I and type IV collagenase for 1 hour and passed through a mesh, after which the erythrocytes were lysed to prepare a single-cell suspension. The collected cells were labeled with specific fluorescence-tagged monoclonal antibodies for CSCs cells and then subjected to flow cytometry analysis.

**2.27 *In vivo* biosafety evaluation in zebrafish**

Zebrafish embryos at the age of 48 hpf were dechorionated with pronase E (Solarbio) and anesthetized with tricaine (Sigma). Zebrafish embryos were randomized into 3 groups (n=20) including the control, AMPM (20 and 200 μg⋅mL^-1^) groups. The Zebrafish embryos were monitored using a fluorescent microscope (SMZ1270, Nikon).

**2.28 Statistical analysis and software**

The data were analyzed via appropriate methods and are presented as the means ± SEMs with respect to the number of samples (n) in each group. Statistical comparisons between two groups were performed via Student’s t test, whereas comparisons among more than two groups were conducted via one-way analysis of variance (ANOVA) via GraphPad Prism software. A p value of *P < 0.05, **P < 0.01, and ***P < 0.001 was considered statistically significant.

**3. Supplementary figures**

**
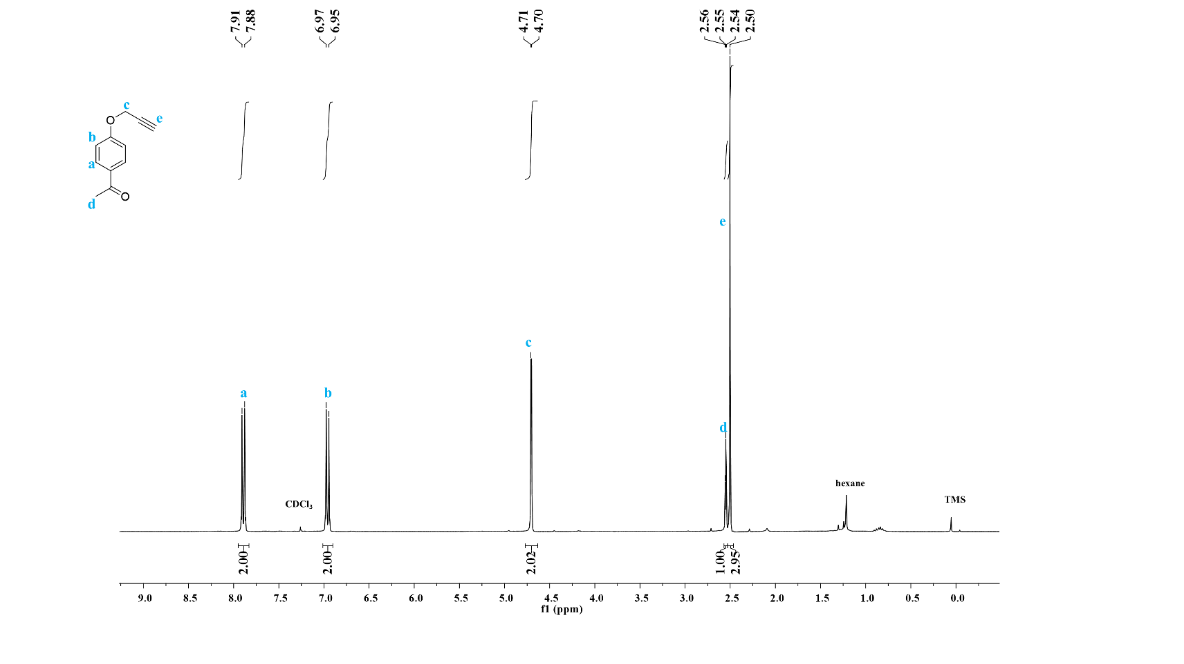
**

**FIGURE S1** ^1^H NMR spectrum (300 MHz) with corresponding assignments and chemical structure of compound **8** in CDCl_3_.


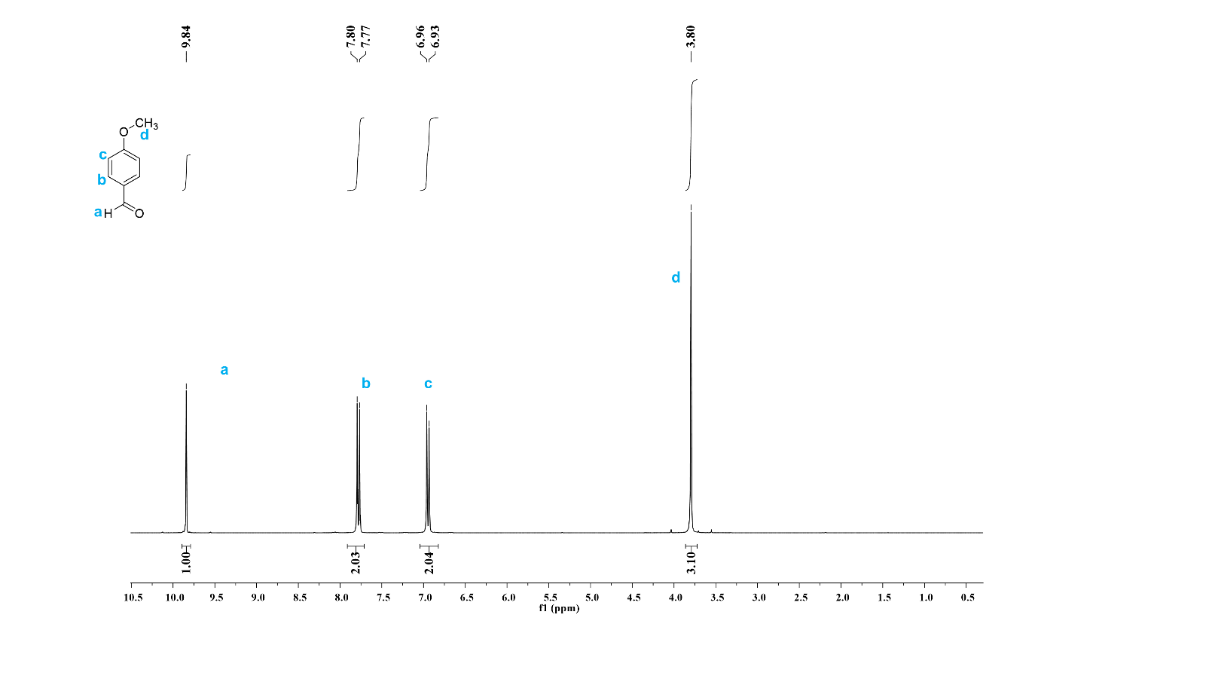


**FIGURE S2** ^1^H NMR spectrum (300 MHz) with corresponding assignments and chemical structure of compound **7** in CDCl_3_.


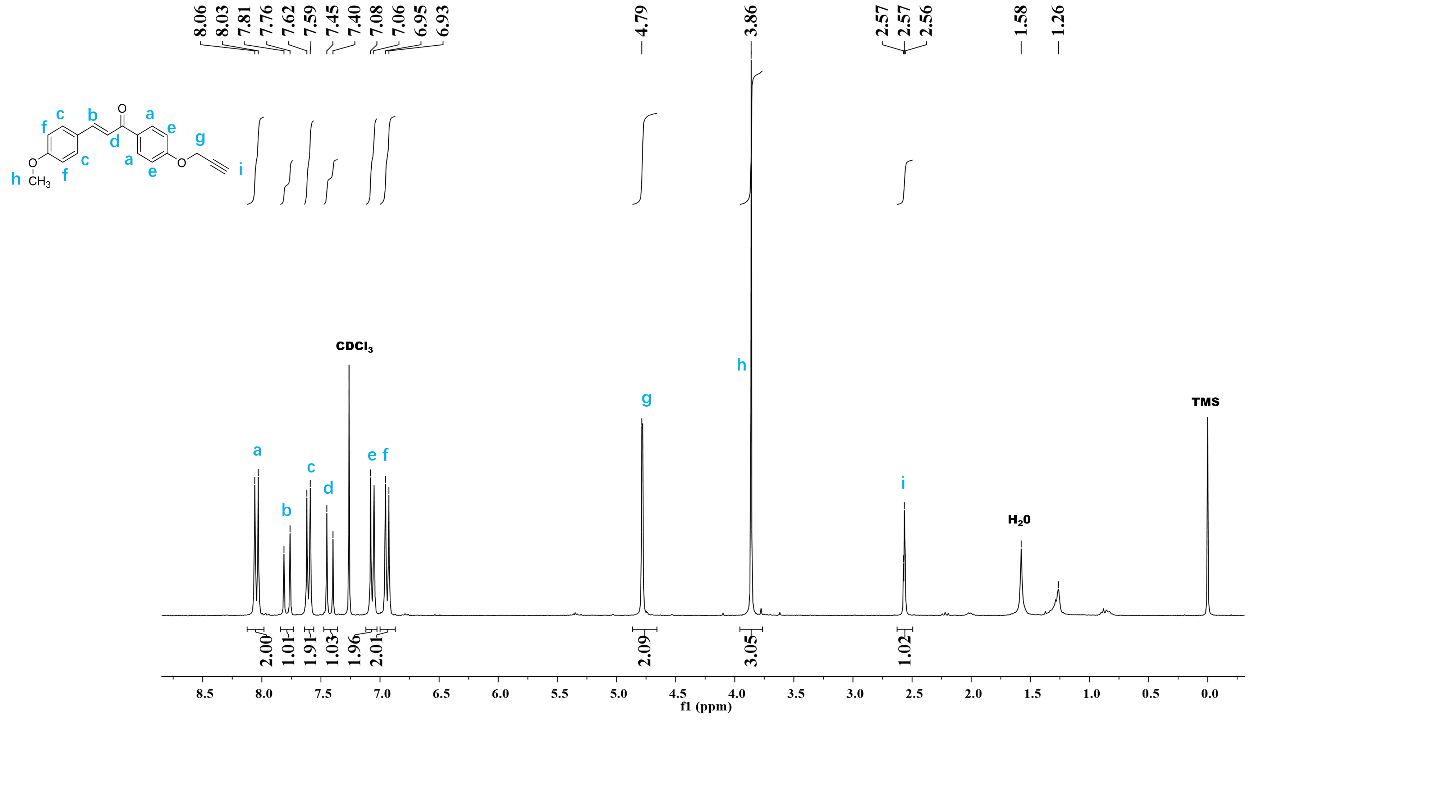


**FIGURE S3** ^1^H NMR spectrum (300 MHz) with corresponding assignments and chemical structure of compound **6** in CDCl_3_.


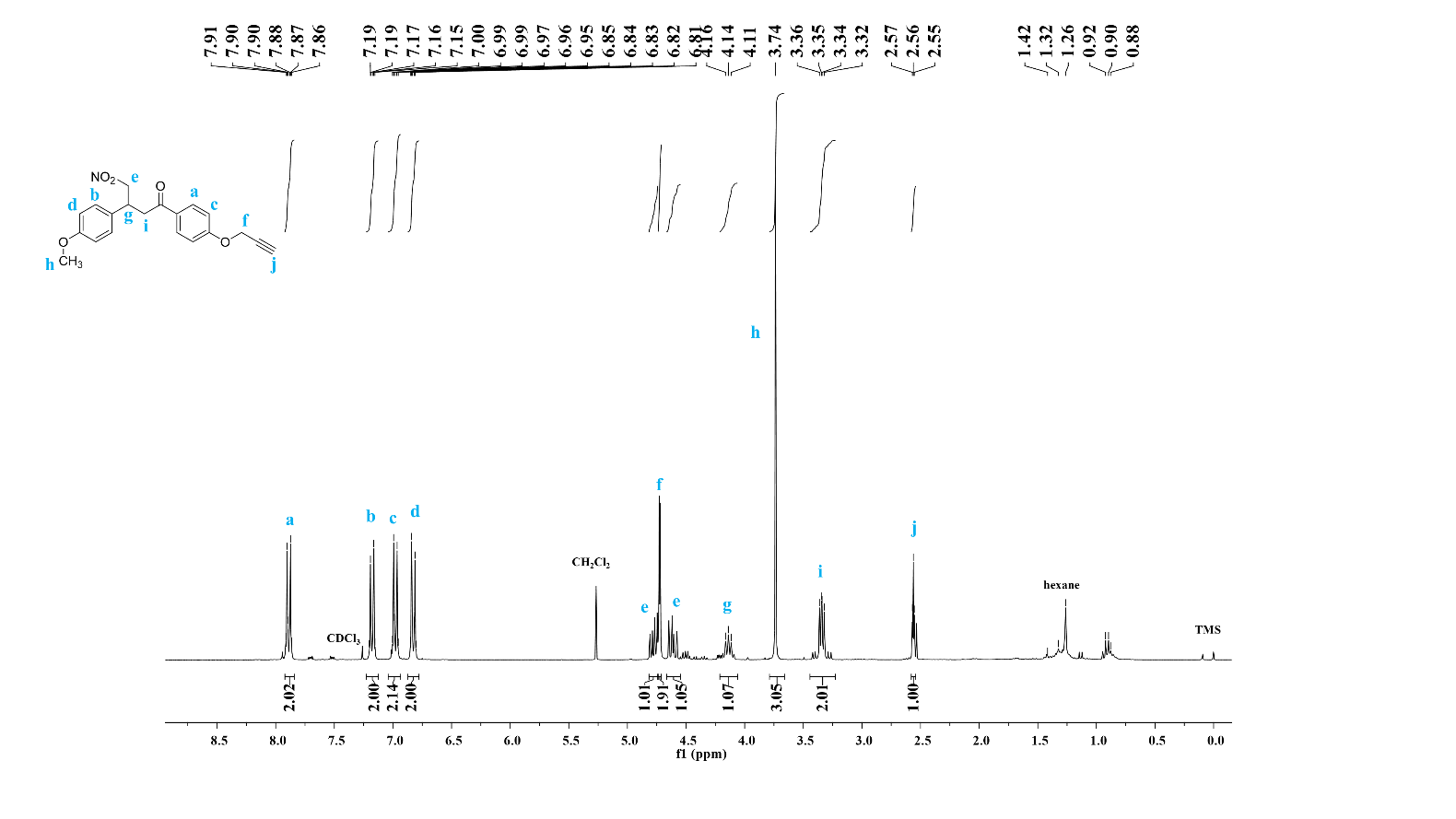


**FIGURE S4** ^1^H NMR spectrum (300 MHz) with corresponding assignments and chemical structure of compound **5** in CDCl_3_.


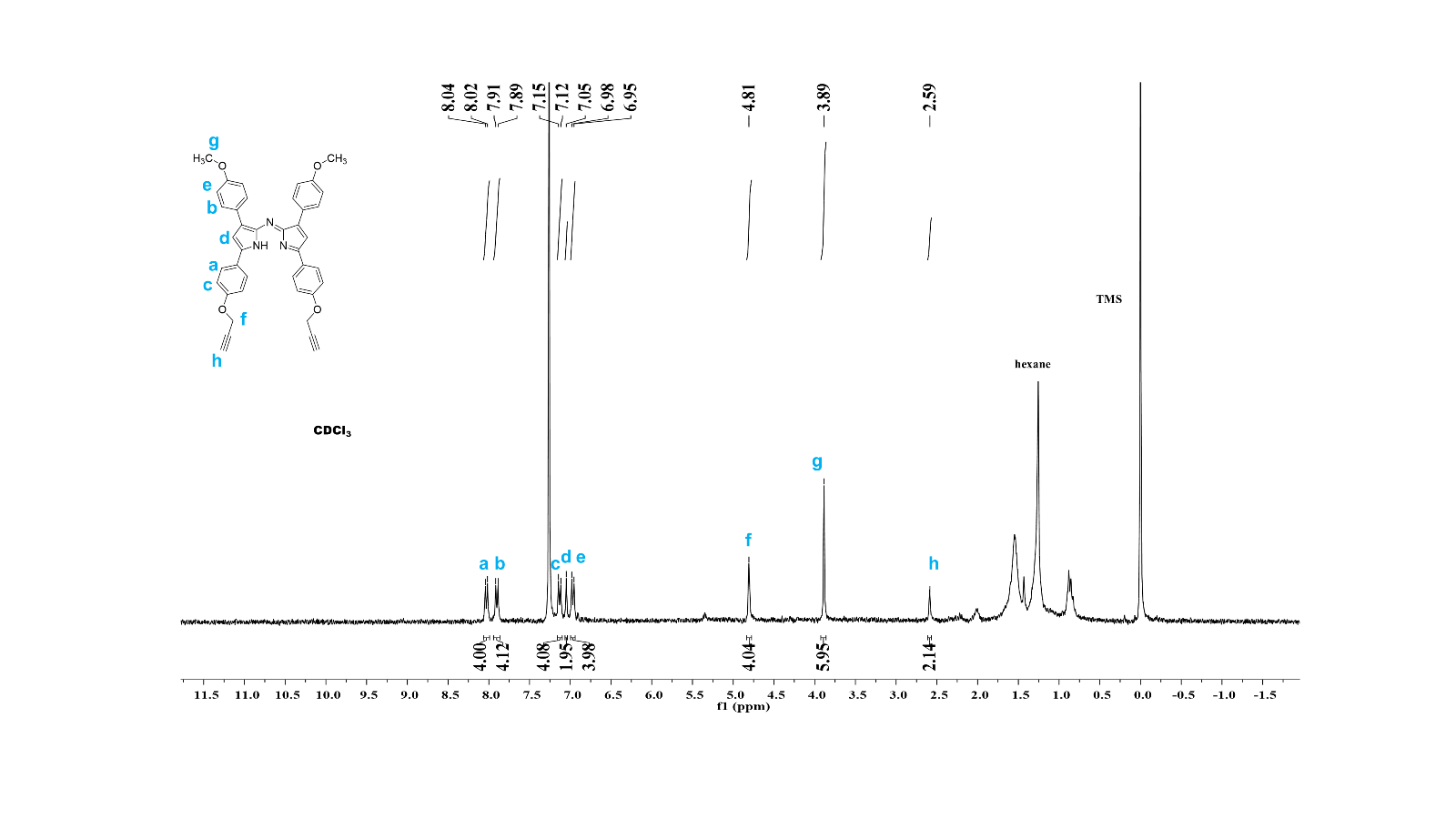


**FIGURE S5** ^1^H NMR spectrum (300 MHz) with corresponding assignments and chemical structure of compound **4** in CDCl_3_.


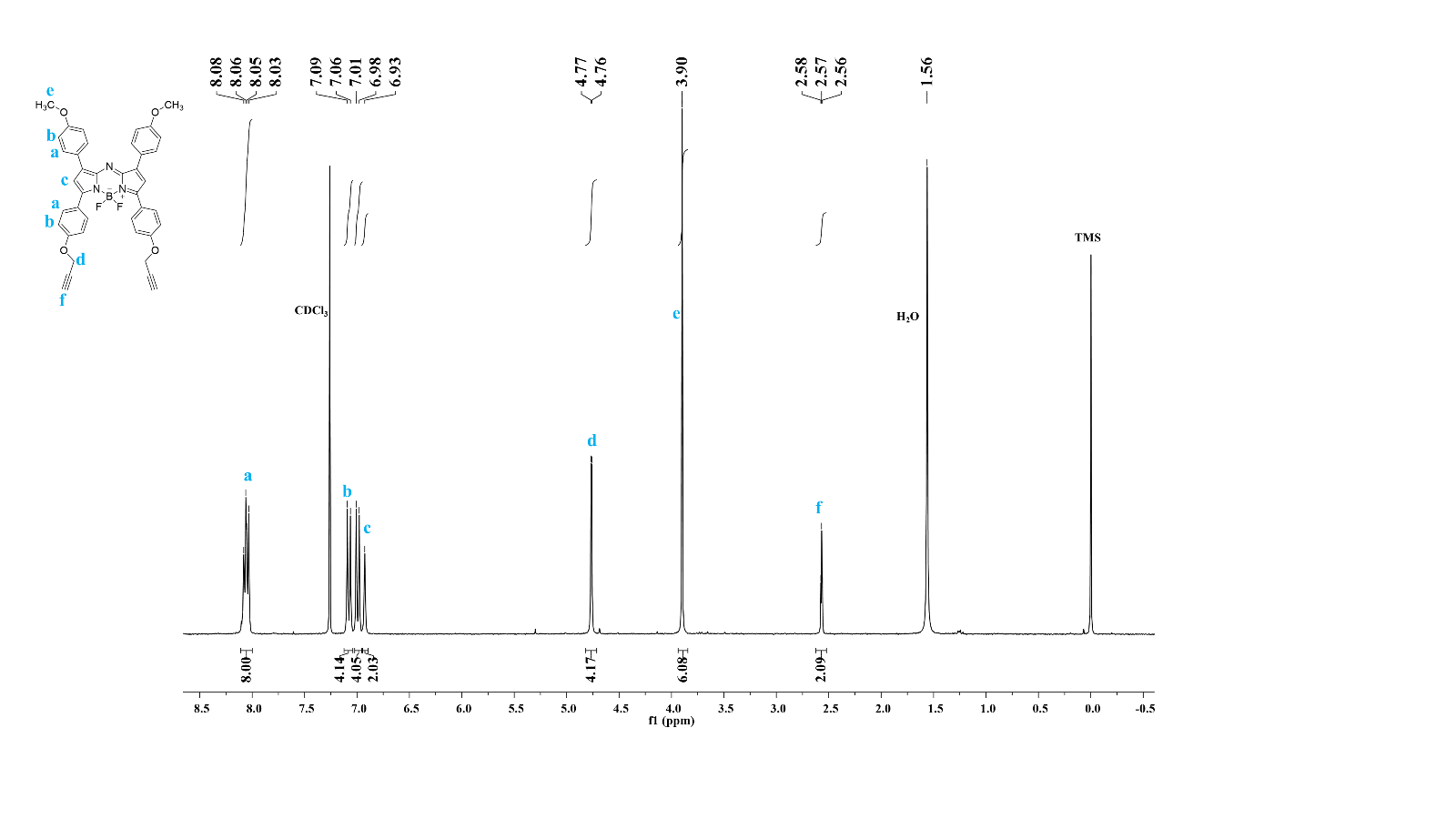


**FIGURE S6** ^1^H NMR spectrum (300 MHz) with corresponding assignments and chemical structure of compound **3** in CDCl_3_.


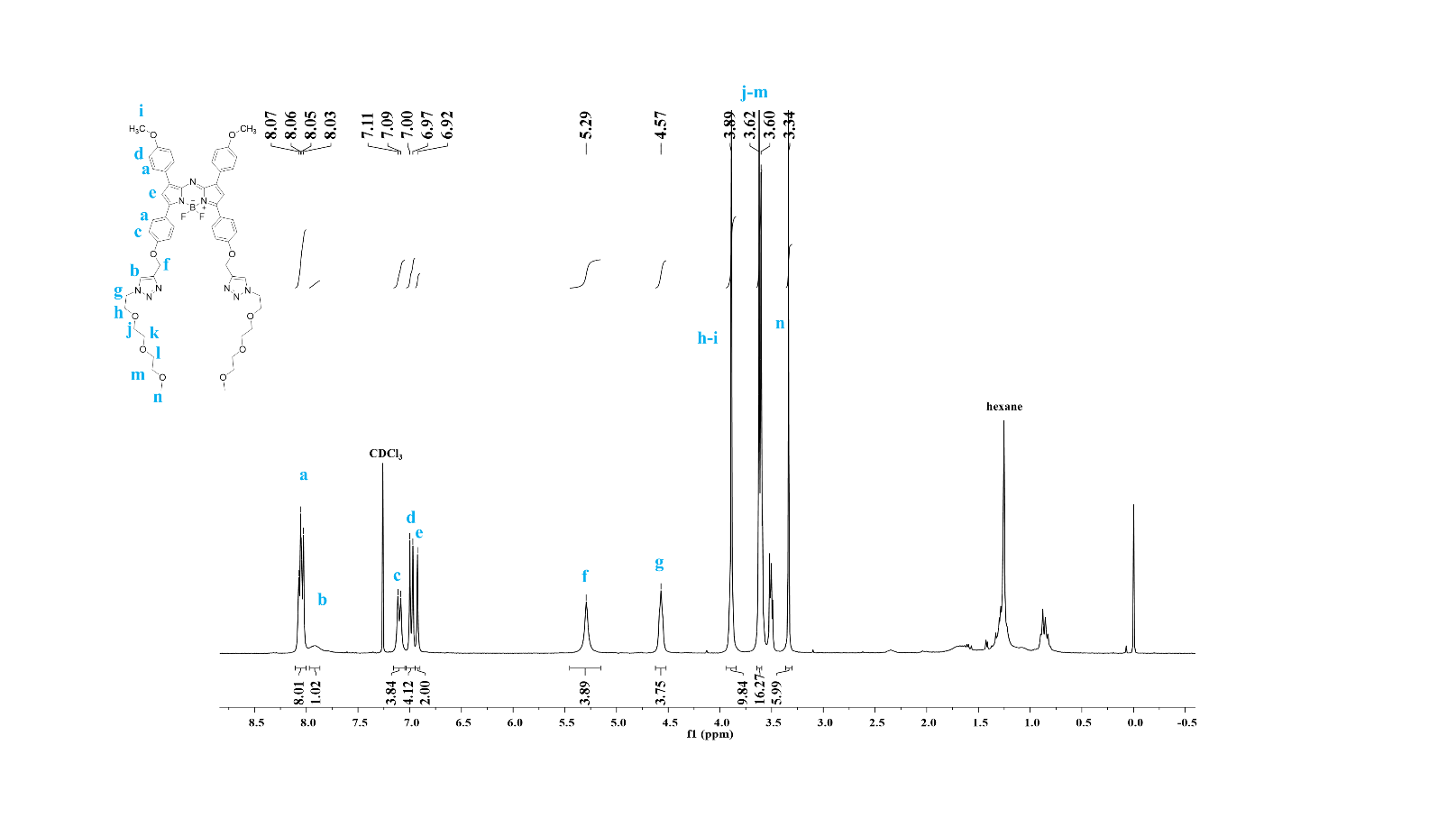


**FIGURE S7** ^1^H NMR spectrum (300 MHz) with corresponding assignments and chemical structure of compound **AB** in CDCl_3_.


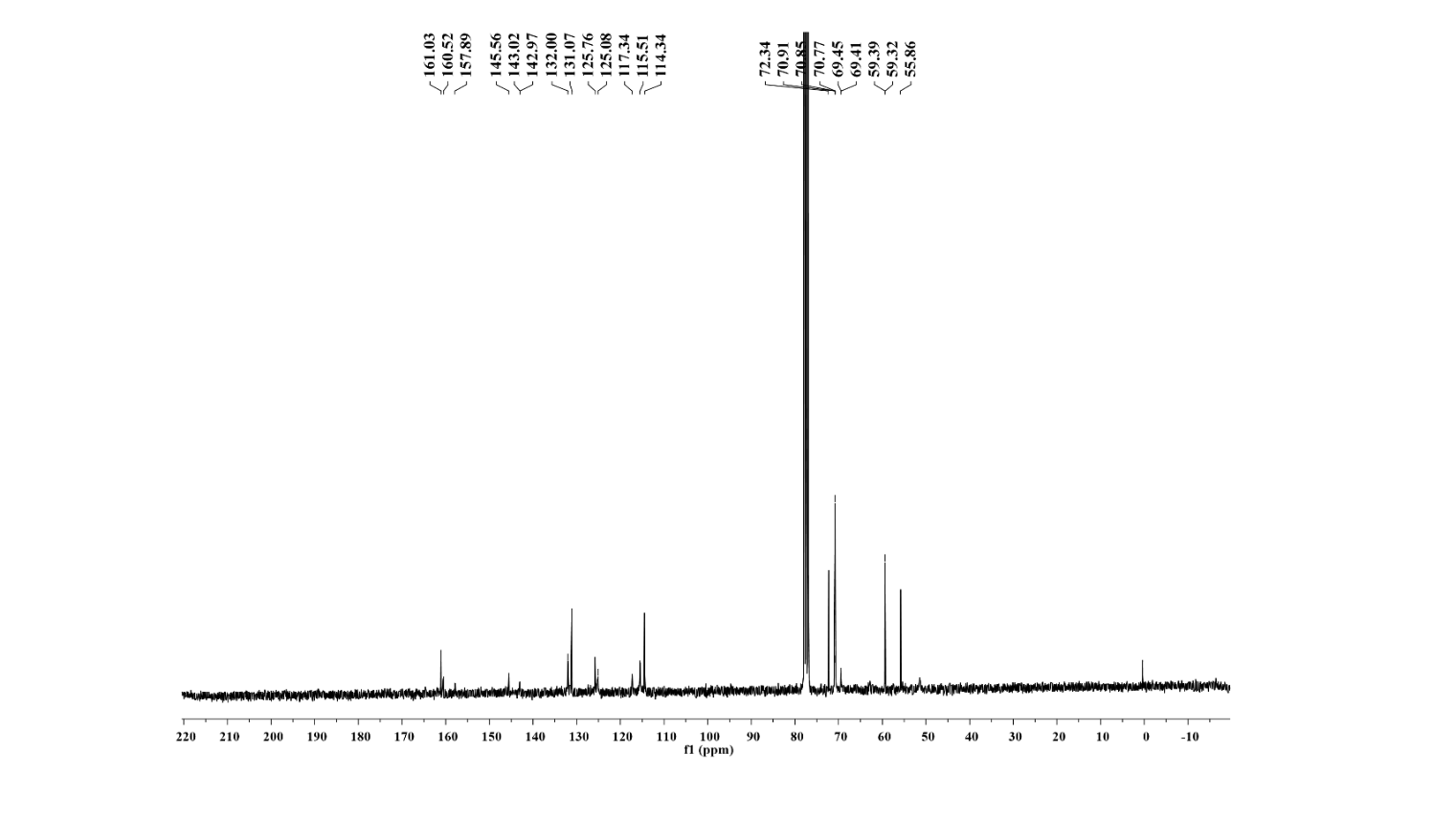


**FIGURE S8** ^13^C NMR spectrum (300 MHz) of compound **AB** in CDCl_3_.

**TABLE S1** List of utilized primer sequences

| Name of Gene | Forward Primer | Reward Primer |
| --- | --- | --- |
| CD44 | GACACATATTGCTTCAATGCTTCAGC | GATGCCAAGATGATCAGCCATTCTGGAA |
| Sox-2 | GCCCTGCAGTACAACTCCAT | GACTTGACCACCGAACCCAT |
| Nanog | GTCCCAAAGGCAAACAACCC | GCTGGGTGGAAGAGAACACA |
| Oct-4 | CTTGAATCCCGAATGGAAAGGG | GTGTATATCCCAGGGTGATCCTC |
| β-actin | GTTGCGTTACACCCTTTCTTG | GACTGCTGTCACCTTCACCGT |


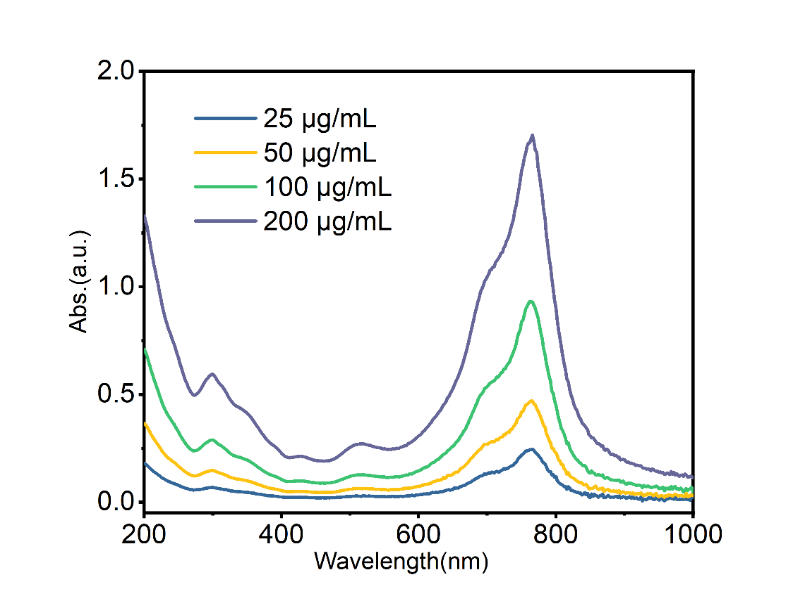


**FIGURE S9** UV/Vis absorbance of different concentrations (25, 50, 100, 200 μg⋅mL^-1^) of AMPM in PBS.


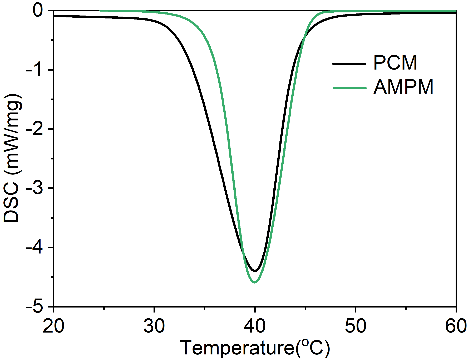


**FIGURE S10** DSC curves of PCM and AMPM.


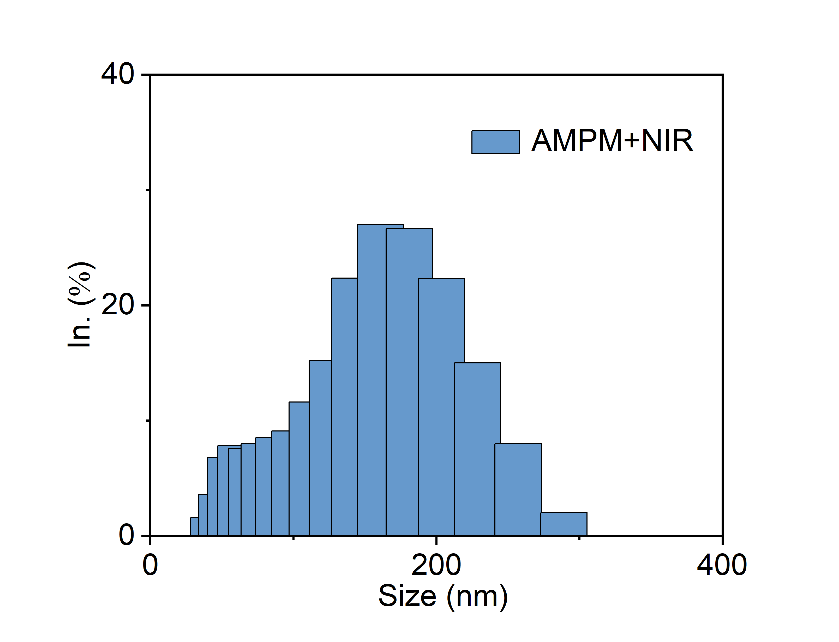


**FIGURE S11** The size distribution of AMPM (50 μg⋅mL^-1^) PBS solution after 5 min irradiation (808 nm, 0.5 W🞄cm^-2^) tested by DLS.


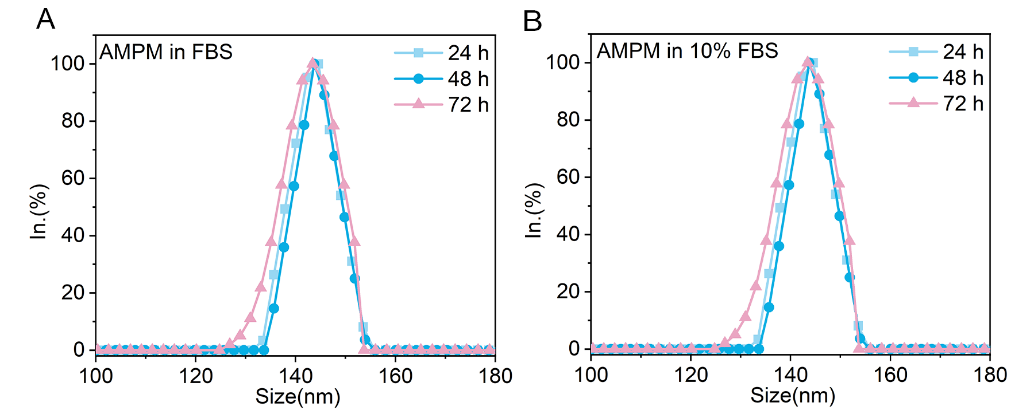


**FIGURE S12** The size distribution of AMPM (50 μg⋅mL^-1^) in PBS (A) and FBS (B) solution at 37 ^o^C tested by DLS.


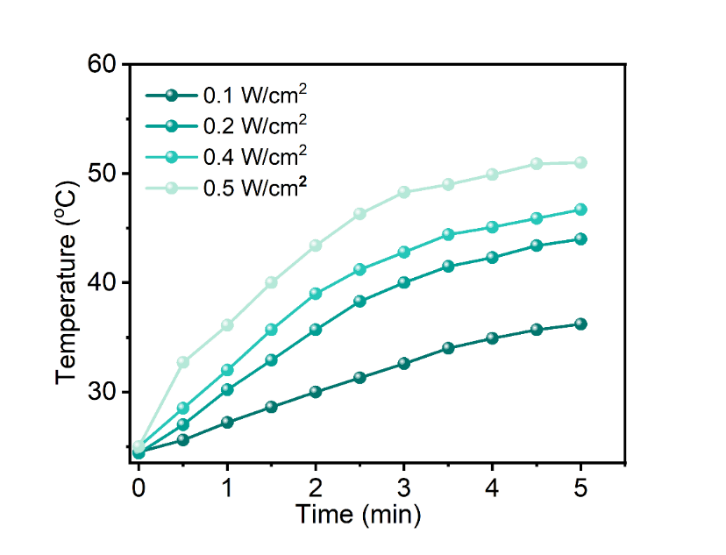


**FIGURE S13** Heating curves of AMPM solution under 808 nm laser irradiation at different power density (0.1, 0.2, 0.4 and 0.5 W⋅cm^-2^) at a total concentration of 100 μg⋅mL^-1^.


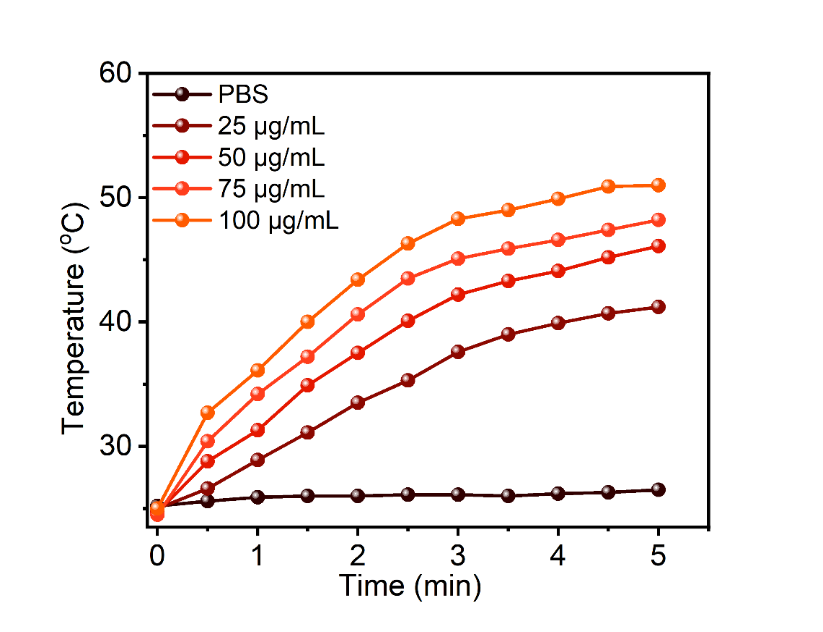


**FIGURE S14** Heating curves of AMPM solution at different concentrations (0, 25, 50, 75 and 100 μg⋅mL^-1^) under 808 nm laser irradiation at a power density of 0.5 W⋅cm^-2^.


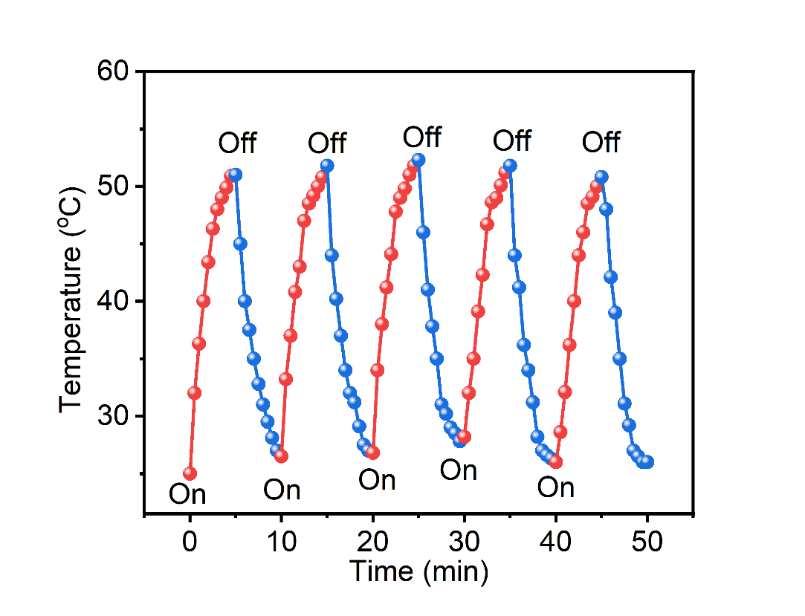


**FIGURE S15** The heating/cooling processes of AMPM were recorded under 808 nm laser irradiation at power density of 0.5 W⋅cm^-2^ at a total concentration of 100 μg⋅mL^-1^.


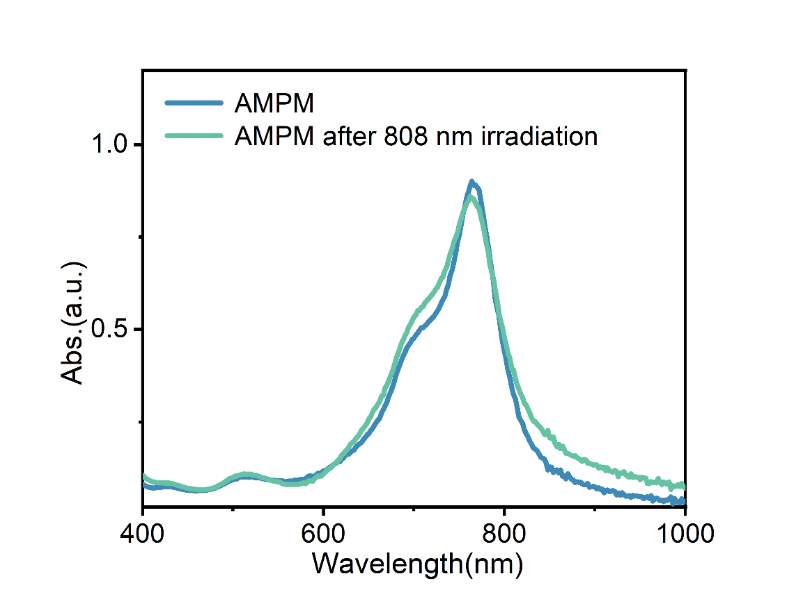


**FIGURE S16** UV/Vis absorption spectra of AMPM before and after irradiation under 808 nm laser irradiation at power density of 0.5 W⋅cm^-2^ at a total concentration of 100 μg⋅mL^-1^.


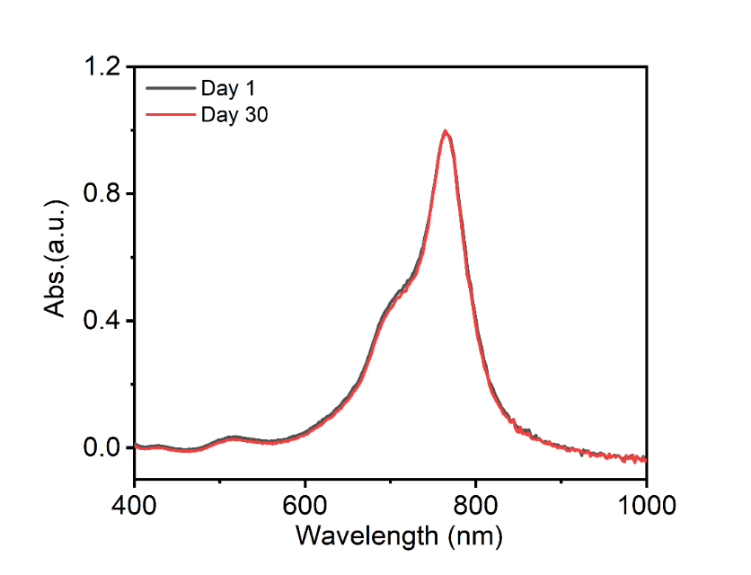


**FIGURE S17** UV/Vis absorbance of AMPP after NIR irradiation (100 μg⋅mL^-1^) in PBS at day 1 and day 30.


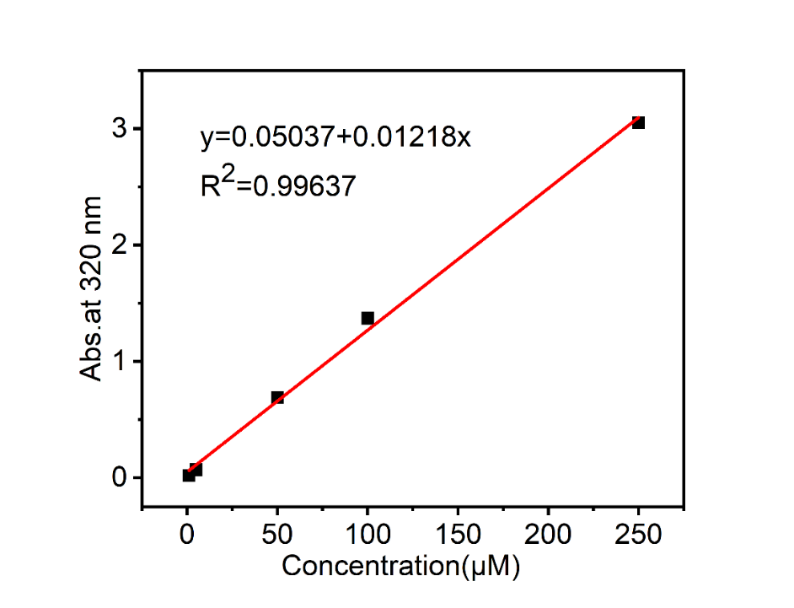


**FIGURE S18** Standard curve of MTZ concentration versus absorbance.


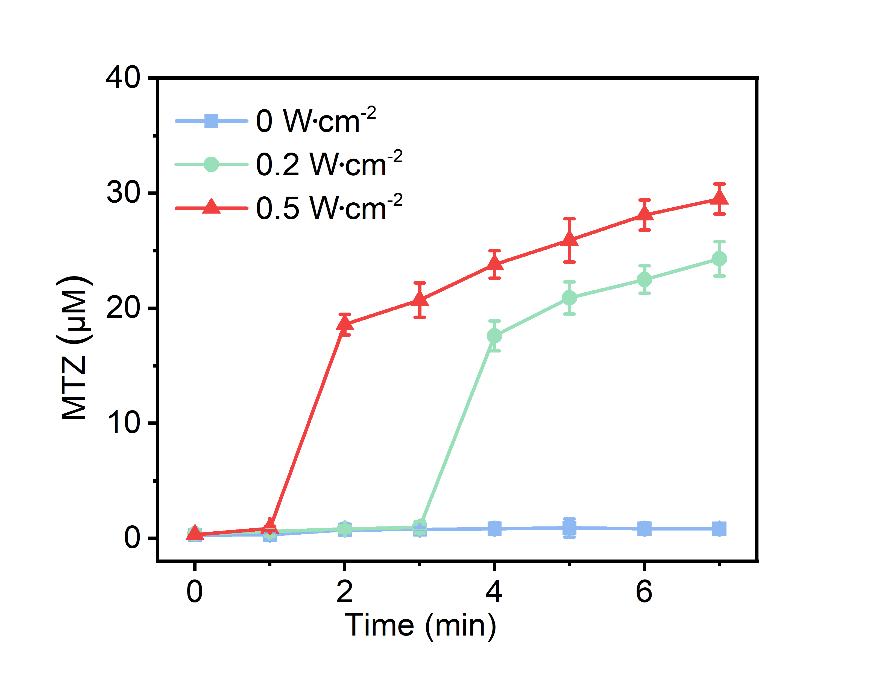


**FIGURE S19** Curves of drug release from AMPM over 7 min at different power densities of 0, 0.2 and 0.5 W⋅cm^-2^ (mean ± SD, n = 3).


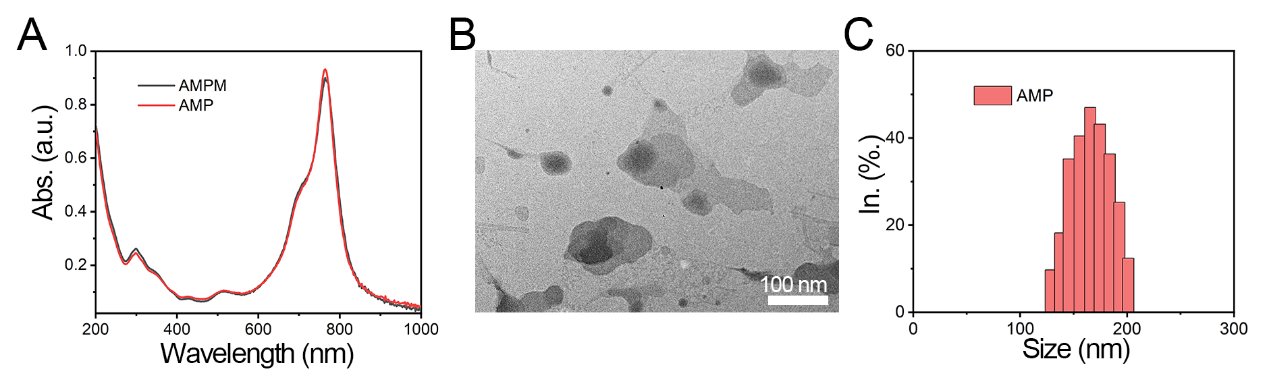


**FIGURE S20** Characterization of AMP. A) UV/Vis absorption spectra of ABM (50 μM, red line) and AMPM (50 μM, black line) in water at room temperature. B) TEM image of AMP. C) The size distribution of AMP (50 μg⋅mL^-1^) PBS solution at 37 ^o^C tested by DLS.


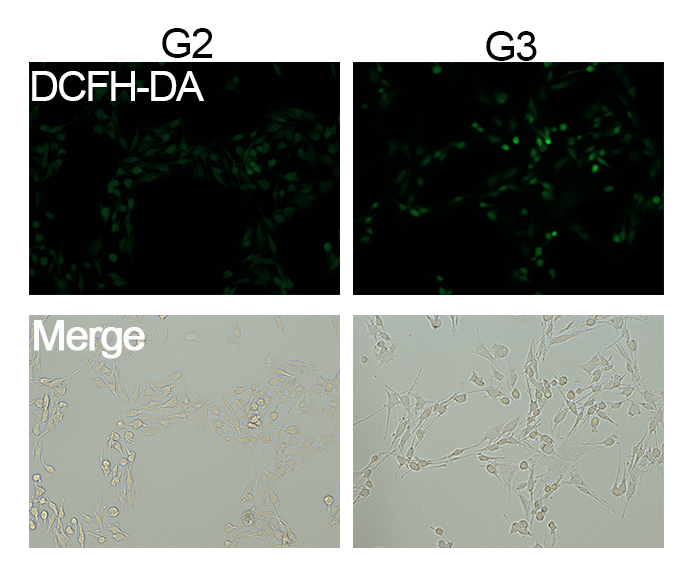


**FIGURE S21** A) Fluorescence image of MDA-MB-231 cells treated with G2 and G3 using DCFH-DA staining (λex = 488 nm, λem = 500‐540 nm).


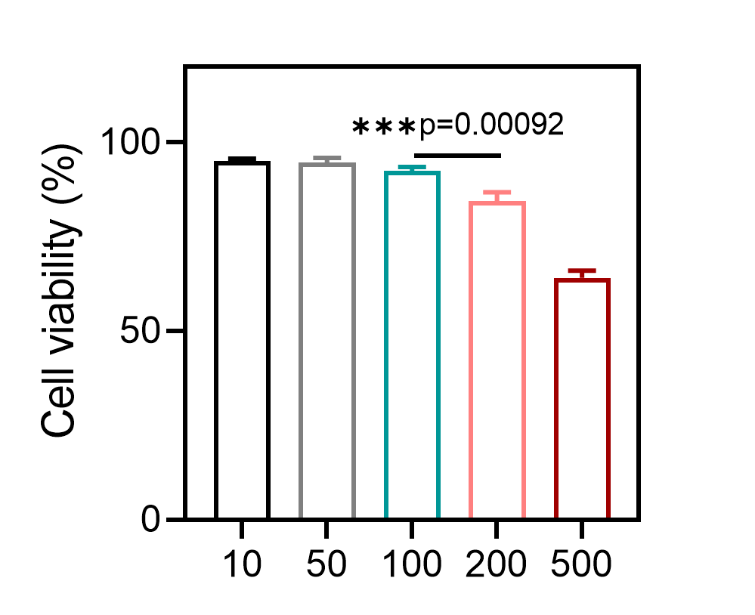


**FIGURE S22** The MDA-MB-231 cell cytotoxicity of AMPM including various PFP (10 μL, 50 μL, 100 μL, 200 μL, 500 μL). Values are expressed as means ± SD (N = 3).


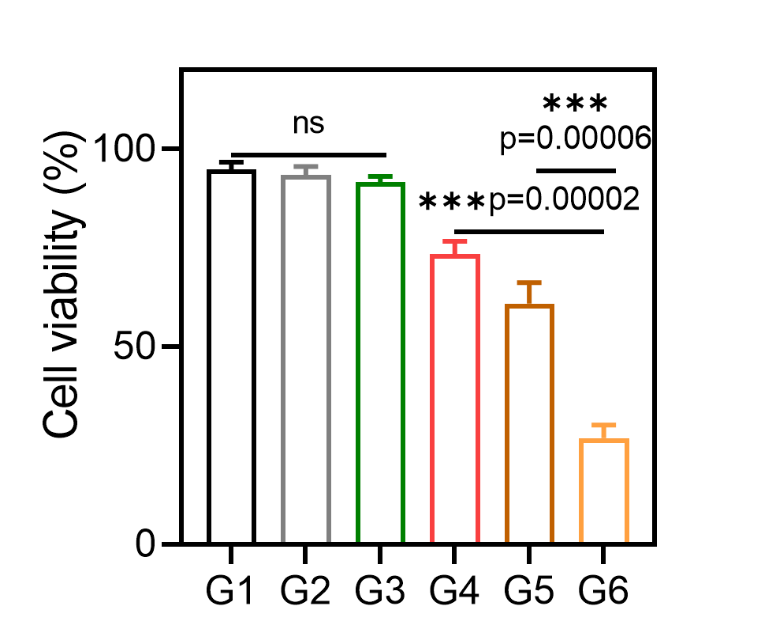


**FIGURE. S23** The MDA-MB-231 cell cytotoxicity treated by various groups. Values are expressed as means ± SD (N = 3).


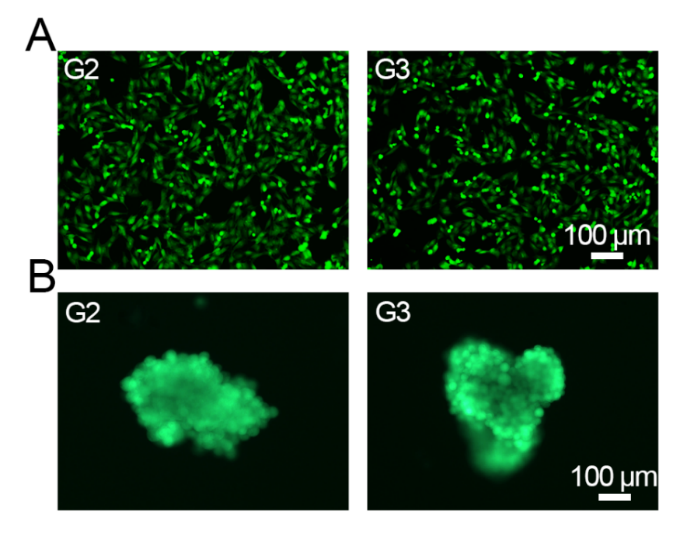


**FIGURE S24** (A) Fluorescence images of MDA-MB-231 cells costained with calcein AM (live cells, green fluorescence) and PI (dead cells, red fluorescence) treated with G2 and G3. (B) Fluorescence images of MDA-MB-231 cells spheroids costained with calcein AM and PI treated with G2 and G3.


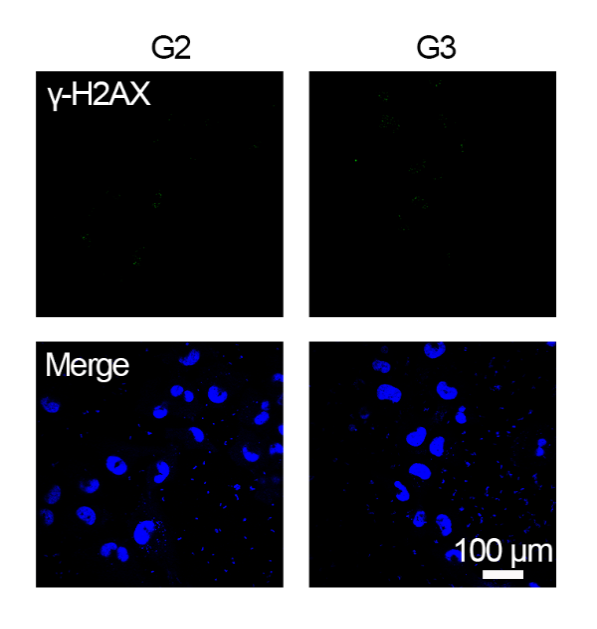


**FIGURE S25** CLSM microscopy observation of γ-H2AX immunofluorescence of MDA-MB-231 cells treated with G2 and G3.


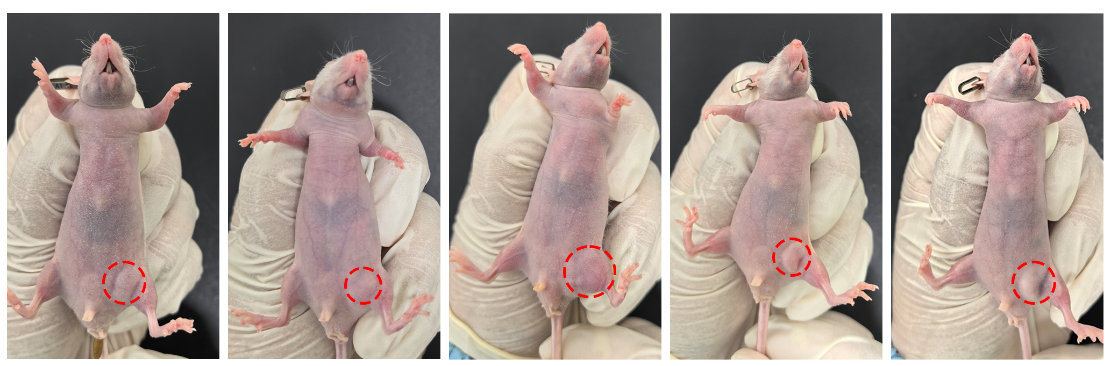


**FIGURE S26** Images of tumor formation on the underarms of nude mice with a density of 1 × 10³ cells during 4 weeks.


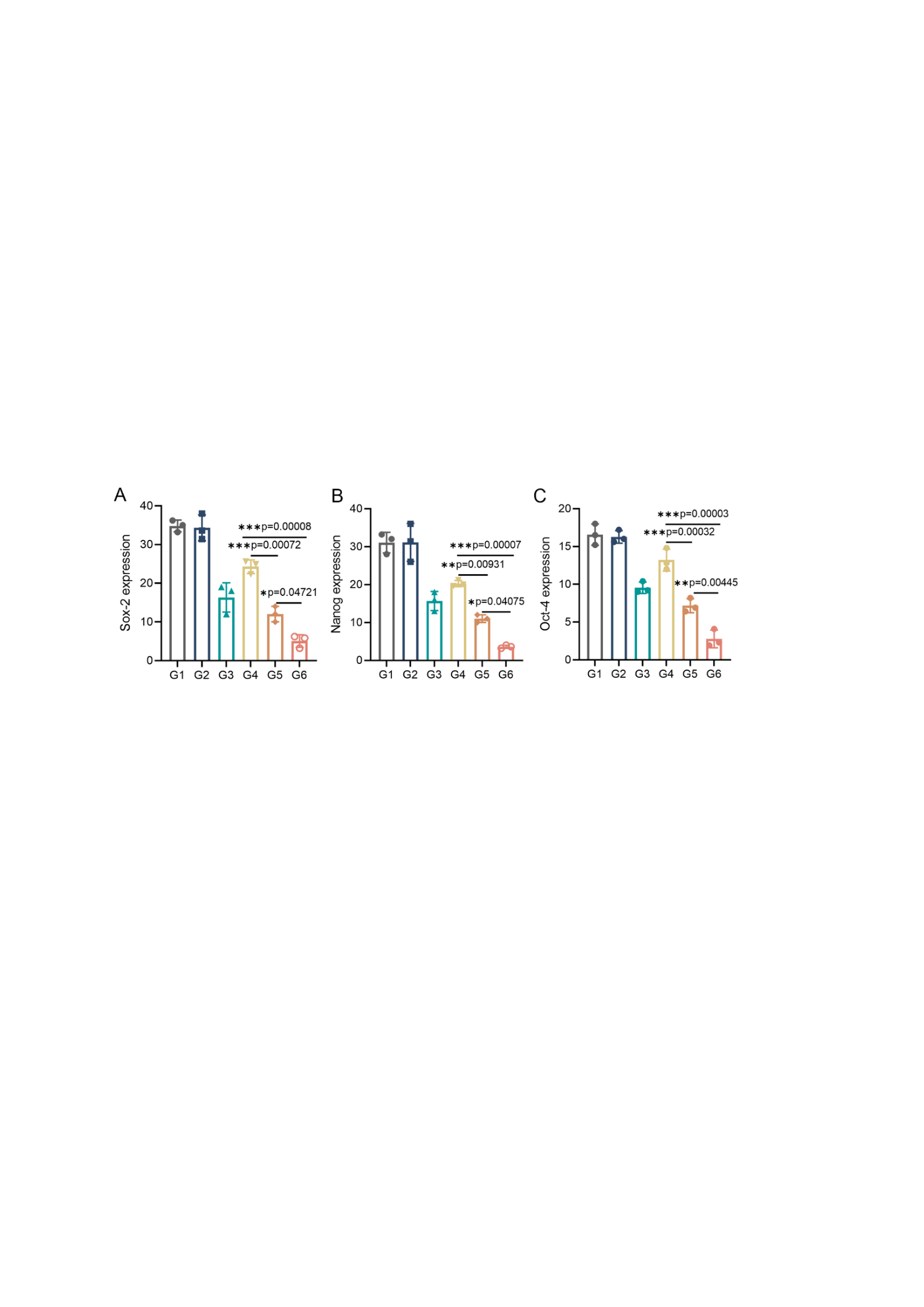


**FIGURE S27** qPCR analysis of Sox-2, Nanog and Oct-4 expression in SP-MDA-MB-231 cells after 24 h in the presence of different.


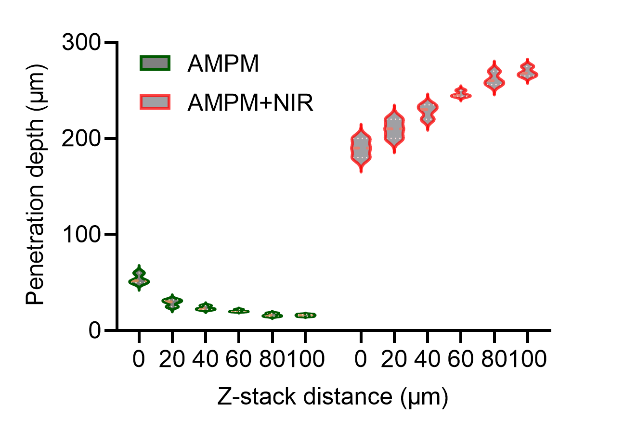


**FIGURE S28** Quantitative measurement of the penetration distance of AMPM into the spheroids without and with NIR irradiation under z-stack scanning conditions.


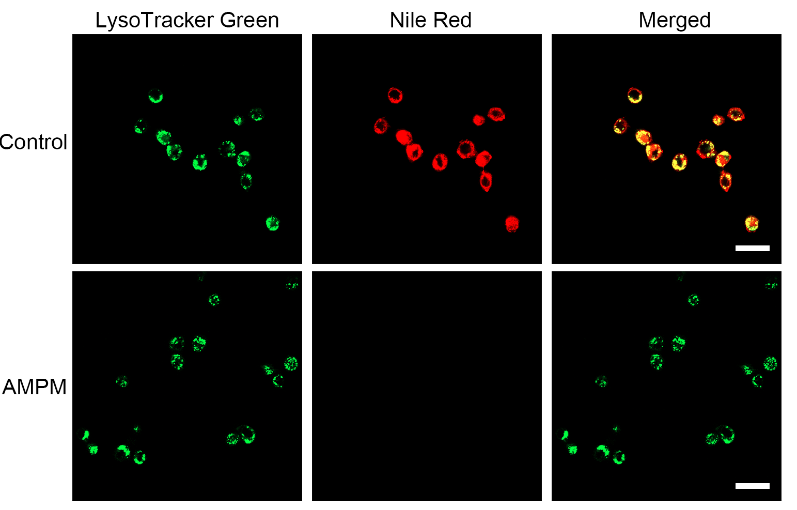


**FIGURE S29** CLSM microscopy observation of MDA-MB-231 cells that were incubated with Nile Red-labeled AMPM (100 μg⋅mL^-1^) for 2 h at 37 ^o^C. Lysosomes were labeled with Lyso Tracker Green DND-26 for 30 min before imaging. Scale bar: 100 μm.


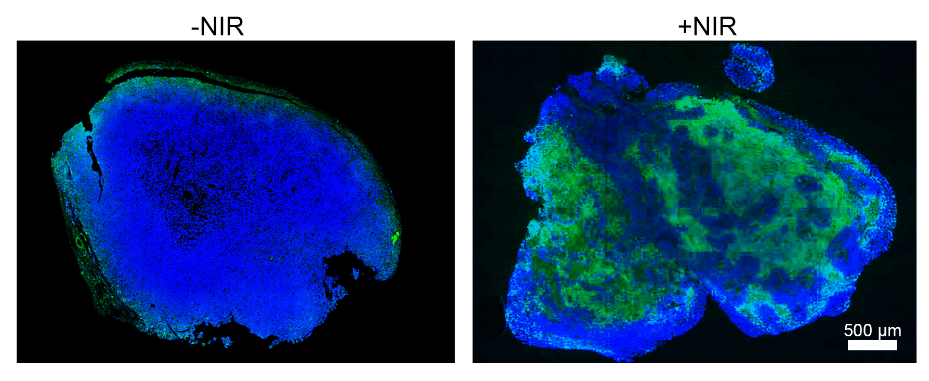


**FIGURE S30** Immunofluorescence images of FITC at tumor sections without and with 808 nm irradiation.


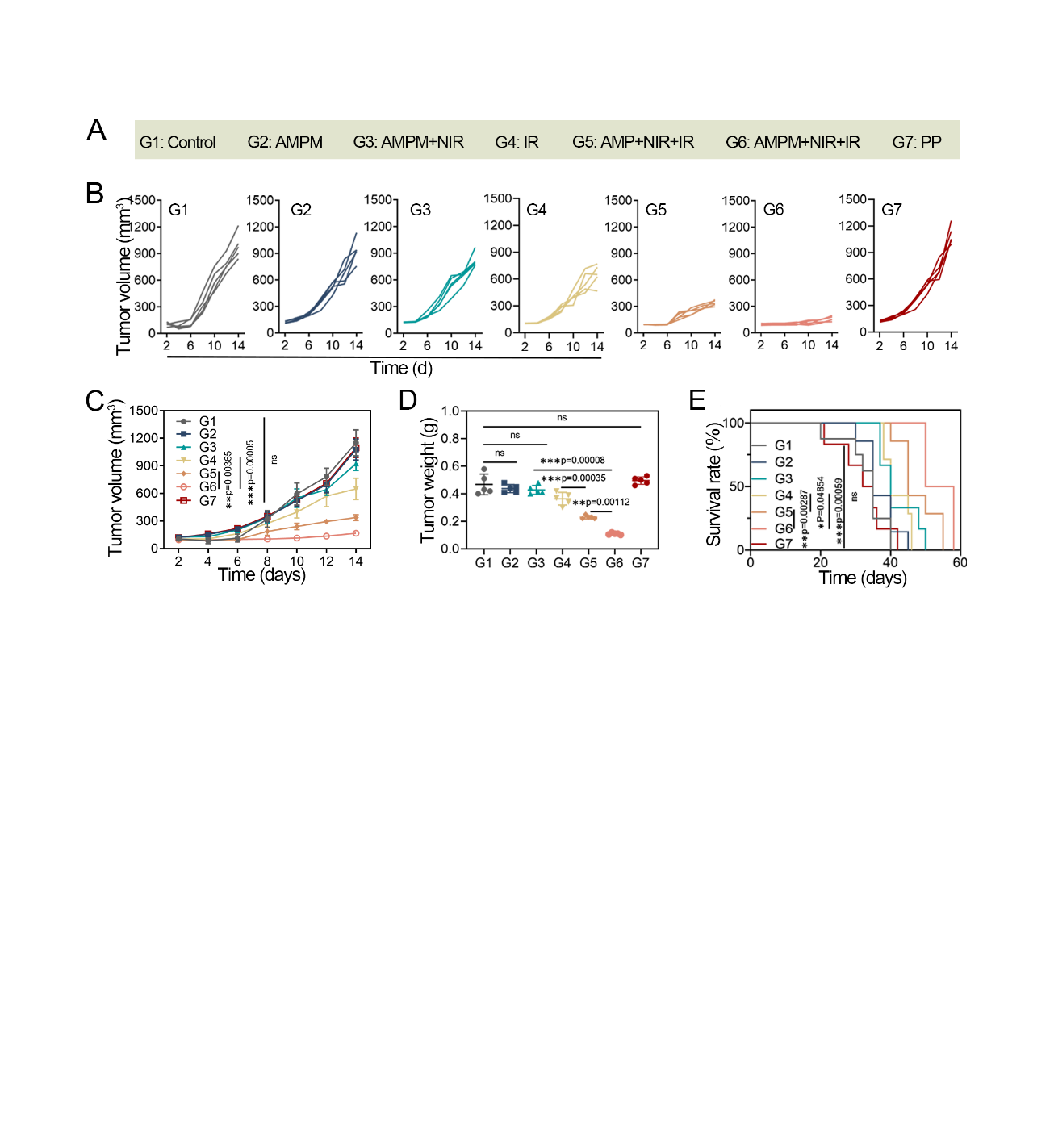


**FIGURE S31** AMPM augments RT against MDA-MB-231 tumor-bearing mice. A) Different treatment groups. B) Individual tumor growth curves of the mice in the different groups. C) Tumor volume curves for the various treatment groups (mean ± SD, n = 5). D) Tumor weight of different groups (mean ± SD, n = 5). E) Kaplan-Meier survival curves of each group (mean ± SD, n = 5). ns = non-significant. A p value of *P < 0.05, **P < 0.01, and ***P < 0.001 was considered statistically significant.


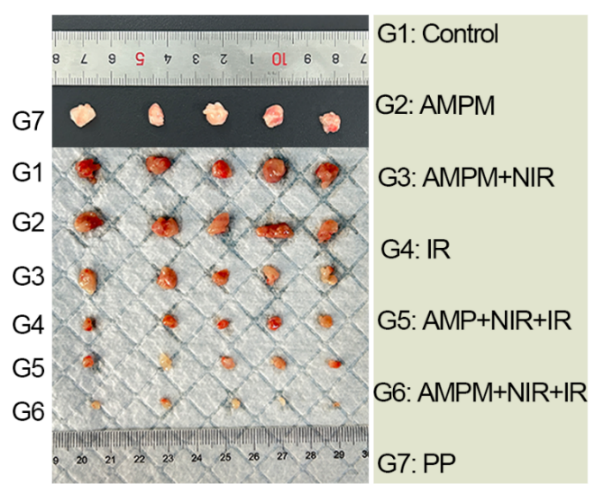


**FIGURE S32** The photographs of the excised tumors from mice.


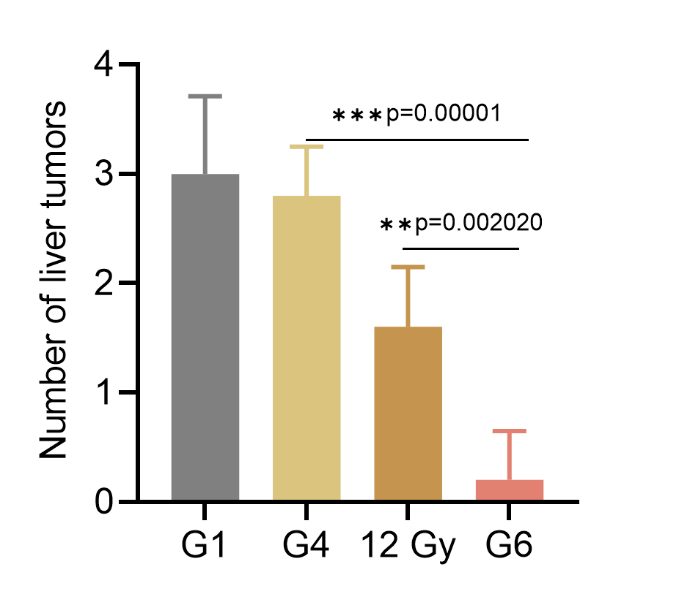


**FIGURE S33** Quantitative graph of the number of liver metastatic tumors in different treatment groups.

**
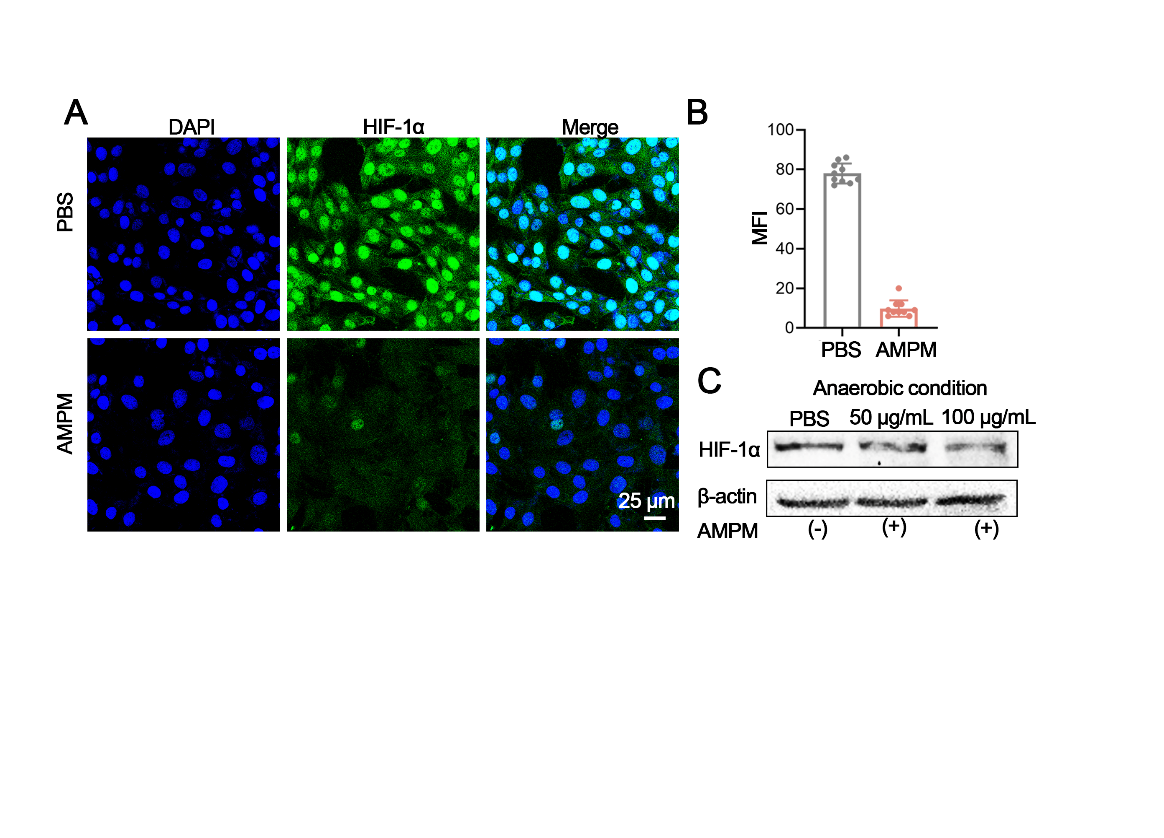
**

**FIGURE S34** A) CLSM microscopy observation of HIF-1α immunofluorescence of MDA-MB-231 cells treated with PBS and AMPM. B) Quantitative analysis of Mean fluorescence intensity from Figure S25A. C) Expression levels of HIF-1α measured by western blotting.


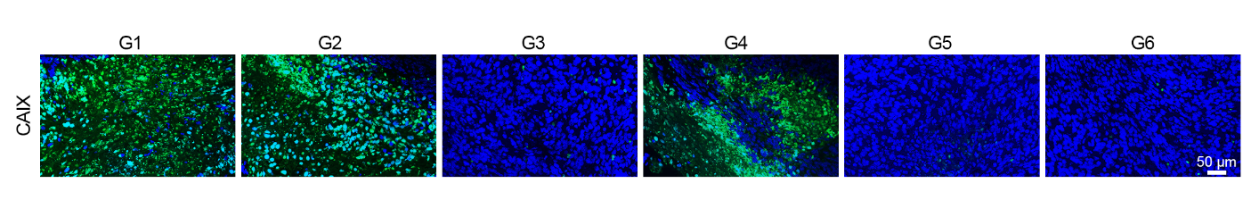


**FIGURE S35** Immunofluorescence images of CAIX at tumor sections after treatment by assigned formulations.


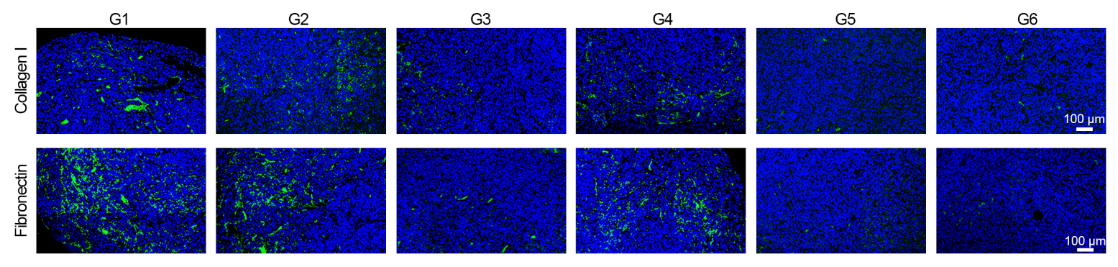


**FIGURE S36** Immunofluorescence images of collagen I, fibronectin at tumor sections after treatment by assigned formulations.


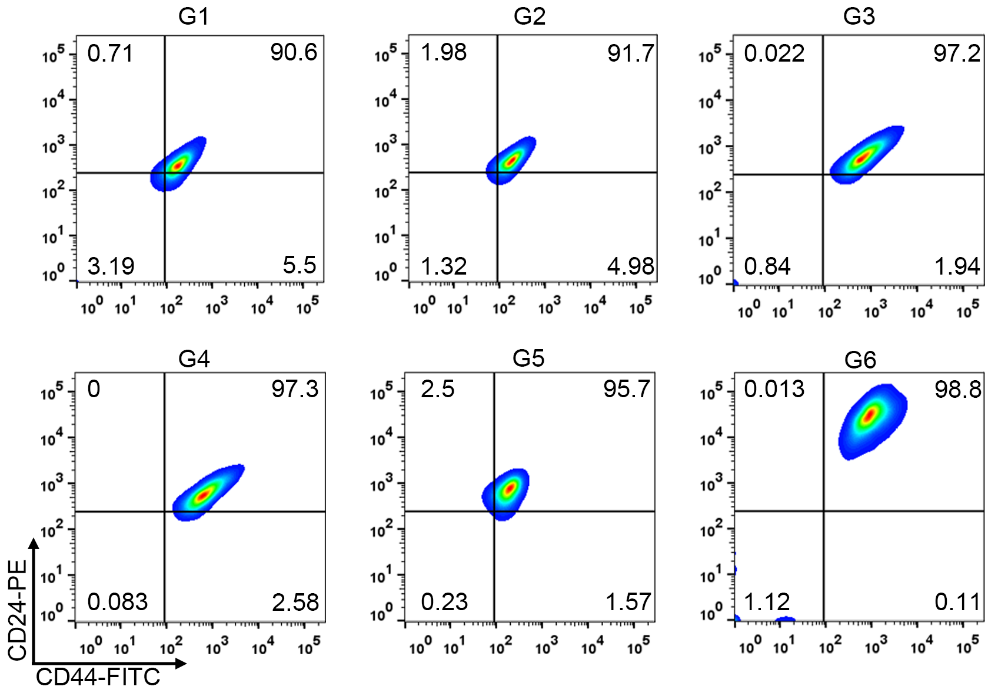


**FIGURE S37** Flow cytometry results for CD44⁺/CD24⁻ cells in tumor tissues in various groups.


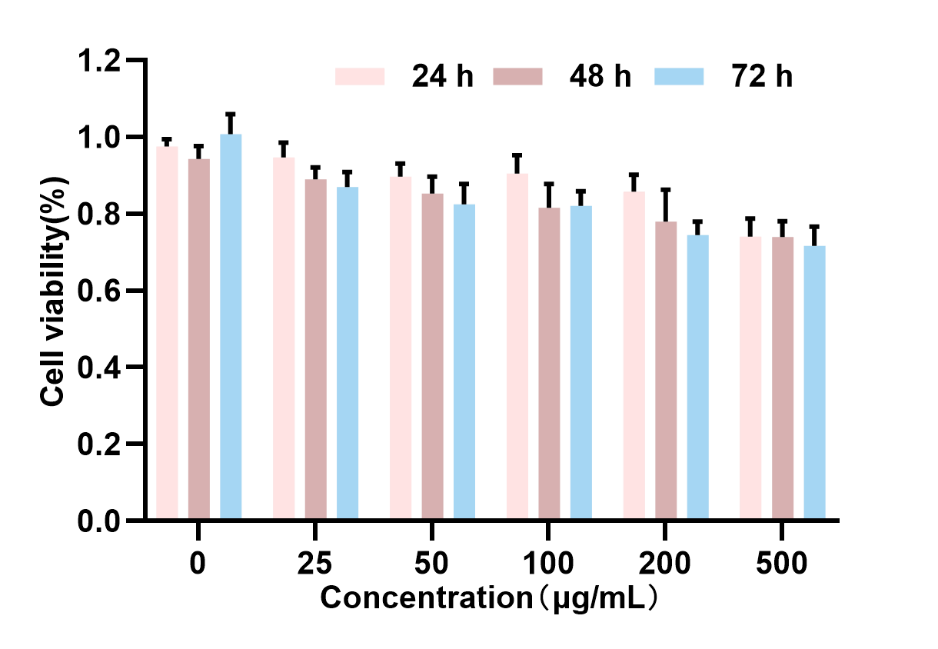


**FIGURE S38** The L929 cell cytotoxicity of AMPM. Values are expressed as means ± SD (N = 3).

**REFERENCES**

[1] Z. Chen, Y. Liu, W. Wagner, V. Stepanenko, X. Ren, S. Ogi, F. Würthner, *Angew. Chem., Int. Ed.* **2017**, 56, 5729.

[2] S. Shen, X. Xu, S. Lin, Y. Zhang, H. Liu, C. Zhang, R. Mo, *Nat. Nanotechnol.* **2021**, 16, 104.
