# Supplementary material for: Phase‐Change Assembling Nanostructures Synergistically Potentiate Tumor Radiosensitivity by Reducing the Stemness of Cancer Stem‐Like Cells
Source: Exploration (Beijing). 2026 Feb 11;6(1):20250179. doi: 10.1002/EXP.20250179 (PMC12970158; doi:10.1002/EXP.20250179)
Supplement: Supplementary file 2 — Supporting File 2: exp270126‐sup‐0002‐tableS1.docx. [file EXP2-6-20250179-s001.docx]

**TABLE S1** List of utilized primer sequences

| Name of Gene | Forward Primer | Reward Primer |
| --- | --- | --- |
| CD44 | GACACATATTGCTTCAATGCTTCAGC | GATGCCAAGATGATCAGCCATTCTGGAA |
| Sox-2 | GCCCTGCAGTACAACTCCAT | GACTTGACCACCGAACCCAT |
| Nanog | GTCCCAAAGGCAAACAACCC | GCTGGGTGGAAGAGAACACA |
| Oct-4 | CTTGAATCCCGAATGGAAAGGG | GTGTATATCCCAGGGTGATCCTC |
| β-actin | GTTGCGTTACACCCTTTCTTG | GACTGCTGTCACCTTCACCGT |
